# Poincaré Plot Area of Gamma-Band EEG as a Measure of Emergence From Inhalational General Anesthesia

**DOI:** 10.3389/fphys.2021.627088

**Published:** 2021-02-09

**Authors:** Kazuma Hayase, Atsushi Kainuma, Koichi Akiyama, Mao Kinoshita, Masayuki Shibasaki, Teiji Sawa

**Affiliations:** Department of Anesthesiology, Kyoto Prefectural University of Medicine, Kyoto, Japan

**Keywords:** anesthesia, electroencephalography, emergence, Poincaré plot, anesthesia depth

## Abstract

The Poincaré plot obtained from electroencephalography (EEG) has been used to evaluate the depth of anesthesia. A standalone EEG Analyzer application was developed; raw EEG signals obtained from a bispectral index (BIS) monitor were analyzed using an on-line monitoring system. Correlations between Poincaré plot parameters and other measurements associated with anesthesia depth were evaluated during emergence from inhalational general anesthesia. Of the participants, 20 were adults anesthetized with sevoflurane (adult__*SEV*_), 20 were adults anesthetized with desflurane (adult__*DES*_), and 20 were pediatric patients anesthetized with sevoflurane (ped__*SEV*_). EEG signals were preprocessed through six bandpass digital filters (f0: 0.5–47 Hz, f1: 0.5–8 Hz, f2: 8–13 Hz, f3: 13–20 Hz, f4: 20–30 Hz, and f5: 30–47 Hz). The Poincaré plot-area ratio (PP_AR_ = PP_*A_fx*_/PP_*A_f0*_, fx = f1∼f5) was analyzed at five frequency ranges. Regardless of the inhalational anesthetic used, there were strong linear correlations between the logarithm of PP_*AR*_ at f5 and BIS (*R*^2^ = 0.67, 0.79, and 0.71, in the adult__*SEV*_, adult__*DES*_, and ped__*SEV*_ groups, respectively). As an additional observation, a part of EMG activity at the gamma range of 30–47 Hz probably influenced the calculations of BIS and PP_*AR_f5*_ with a non-negligible level. The logarithm of PP_AR_ in the gamma band was most sensitive to state changes during the emergence process and could provide a new non-proprietary parameter that correlates with changes in BIS during measurement of anesthesia depth.

## Introduction

The need to maintain a proper depth of general anesthesia (GA) during surgery is an important aspect of anesthesiology. An underdose of anesthetics increases the risk of intraoperative awareness, which may cause long-term psychological problems for the patient (Leslie et al., 2010; Bischoff and Rundshagen, 2011). An overdose of anesthetics may lead to postoperative neurocognitive dysfunction (Cole and Kharasch, 2018). Among various technologies used to optimize the depth of anesthesia, indices for monitoring processed electroencephalograms (EEG), such as bispectral index (BIS), and patient state index (PSI), are popular and have been used in various clinical studies (Fahy and Chau, 2018). Although these anesthesia depth monitoring methods are not standardized, their clinical usefulness is unquestionable as they are widely used by anesthesiologists worldwide. However, most of these devices use proprietary algorithms to measure the depth of anesthesia; moreover, clinical studies using these monitors have demonstrated controversial outcomes. Some trials reported that a BIS protocol reduced the incidence of intraoperative awareness (Ekman et al., 2004; Myles et al., 2004), while others failed to show the superiority of BIS for prevention of intraoperative awareness (Avidan et al., 2008, 2011; Lewis et al., 2019). Similarly, several clinical studies have concluded that the advantages of EEG-guided management include a lower incidence of delirium (Chan et al., 2013; Radtke et al., 2013; Luo and Zou, 2018; MacKenzie et al., 2018), whereas more recent studies failed to show the superiority of EEG-guided management (Wildes et al., 2019; Tang et al., 2020). These controversial outcomes have been explained by several factors (Abbott and Pearse, 2019; Whitlock and Avidan, 2020). Carrying out research using parameters for which the calculation algorithms are unknown raises concerns that simple comparisons cannot be performed among studies. Thus, there is a need to develop a multifaceted and non-proprietary algorithm for the evaluation of anesthesia depth.

There are several notable research approaches regarding the quantification of anesthesia depth, such as cardiorespiratory interactions in distinguishing awake from anesthetized states (Musizza et al., 2007; Kenwright et al., 2015), anesthesia-induced alterations of functional connectivity across the cortex (potentially important for both consciousness and anesthesia) (Chang et al., 2016; Li et al., 2019), and recurrence quantification analysis (Becker et al., 2010). Also, various methods based on entropy analysis have been reported (Bruhn et al., 2000b; Jiang et al., 2015; Su et al., 2016). Some of them seem useful for identifying burst suppression observed in the deep anesthesia phase (Liang et al., 2015). On the other hand, few reports of EEG parameters show the effectiveness in the shallow anesthesia phase leading to the arousal stage. As the BIS monitor combines multiple algorithms to calculate the BIS value for anesthesia depth, it is unlikely that there will be a universal index with a single EEG parameter covering all anesthesia phases at the different anesthesia depth levels.

One approach involves the Poincaré plot, which has been used to analyze various physiological signals (Tulppo et al., 1996; Carrasco et al., 2001; Golińska, 2013). The Poincaré plot is a type of recurrence plot used to quantify self-similarity in processes, usually periodic functions; it can be used to distinguish chaos from randomness by embedding a dataset into a higher-dimensional state space (Golińska, 2013). The Poincaré plot generated from EEG signals has shown correlations with the spectral edge frequency below which 95% of a given signal’s power (SEF_95_) is located during inhalational anesthesia (Hayashi et al., 2015a,b). However, this correlation between SEF_95_ and Poincaré plot indices, similar to the correlation between BIS and SEF_95_, was observed only when the depth of anesthesia is somewhat deep and dissociated during the awakening process from GA. Therefore, in this study, we focused on the emergence from GA, and we thought it is crucial to analyze the higher frequency band of EEG to capture the arousal state. We applied Poincaré plot analysis to processed EEG signals with finite impulse response (FIR) bandpass filters to investigate systematic changes to the Poincaré plot during GA. The Poincaré plot analysis applied to the bandpass-filtered EEG is positioned between time- and frequency domain analysis. We think it is a new method that may incorporate time- and frequency-domain analysis characteristics in univariate time-series analysis. Here, we report that the Poincaré plot-area ratio (PP_*AR*_) of gamma-band EEG constitutes a new independent parameter with sensitivity for state changes from anesthesia to arousal during the emergence process.

## Materials and Methods

### Anesthesia Management and Data Acquisition

All experiment protocols involving humans were conducted in accordance with the principles of the Declaration of Helsinki. The current study was approved (No. ERB-C-1074-2) by the Institutional Review Board for Human Experiments at the Kyoto Prefectural University of Medicine (IRB of KPUM), and for this non-interventional and noninvasive retrospective observational study, informed patient consent was waived by the IRB of KPUM; patients were provided an opt-out option, of which they were notified in the preoperative anesthesia clinic. In our facility, the use of a BIS monitor is routine for adult and pediatric patients who undergo surgery involving GA. The anesthesiologists in charge of management did not receive notice of the study and planned the anesthesia methods for scheduled surgeries in accordance with our facility’s standard care protocol, without any feedback regarding the on-line analysis of processed EEG signals. Patients were not premedicated before anesthesia induction, in accordance with our facility’s standard protocol. An anesthetic gas monitor (IntelliVue G5 Anesthesia Gas Module, Philips, Amsterdam, Netherlands) was used for the measurement of end-tidal sevoflurane (et_*SEV*_) and desflurane (et_*DES*_) concentrations. The end-tidal anesthetic gas concentration (et_*AG*_) was automatically recorded at 1-min intervals on a data server, then retrieved after the completion of anesthesia management. Based on the et_*AG*_ data for each minute, et_*AG*_ values at 20-s intervals were obtained by spline interpolation. In pediatric patients, GA was induced with an inhalational mixed gas, whereas in adult patients, rapid induction with propofol was employed (because of the irritating effects of desflurane on the respiratory tract during slow induction, all anesthesiologists in our facility refrain from the use of desflurane in children). After anesthesia induction, rocuronium (0.8–1.0 mg × kg^–1^) was intravenously administered, and tracheal intubation was conducted. The anesthesia was maintained with approximately 2.5% sevoflurane or 6% desflurane, small doses of fentanyl (1 μg × kg^–1^ per dose), and additional maintenance doses rocuronium (0.2 mg × kg^–1^ at intervals of 20–30 min). The timing of sugammadex administration was significantly earlier in the adult__*SEV*_ group than in the adult__*DES*_ group (*p <* 0.05) (Table 1). The timing of the end of surgery was significantly earlier in the adult__*SEV*_ group than in either adult__*DES*_ (*p <* 0.05) or ped__*SEV*_ groups (*p <* 0.01) (Table 1). In this EEG analysis, we focused on the 10-min period beginning around the end of surgery and ending with emergence from anesthesia; the study of EEG during the emergence process is important for preventing accidental intraoperative awakening. We analyzed the changes in correlations over time of various Poincaré plot parameters with et_*AG*_, EEG parameters such as observed BIS, SEF_95_, and total power (TP), and electromyography (EMG) parameter EMGlow.

**TABLE 1:** Patient characteristics and anesthesia management parameters.

**Item/group**	**Adult__*SEV*_ (*n* = 20)**	**Adult__*DES*_ (*n* = 20)**	**Ped__*SEV*_ (*n* = 20)**	**all__*combined*_ (*n* = 60)**	**ANOVA *p***
Age	56.5 ± 16.8 [48.6–64.4]	55.1 ± 13.7** [48.6–61.5]	4.6 ± 2.7‡ [3.3–5.8]	38.7 ± 27.3‡ [31.6–45.8]	<0.0001
Gender, male/female	8/12	7/13	14/6	29/31	0.1250^§^
Height (cm)	161.9 ± 9.7 [157.5–166.5]	162.5 ± 8.9 [158.4–166.7]	105.2 ± 20.2‡ [95.7–114.6]	143.2 ± 30.4‡ [7135.4–151.1]	<0.0001
Weight (kg)	61.5 ± 11.2 [56.3–66.7]	62.6 ± 11.5 [57.3–68.0]	18.1 ± 7.1‡ [14.7–21.5]	47.4 ± 23.2‡ [41.4–53.4]	<0.0001
Surgery time (min)	97.6 ± 42.0 [78.0–117.3]	114.1 ± 55.6 [88.1–140.1]	74.6 ± 35.9 [57.8–91.4]	95.4 ± 47.4 [83.2–107.7]	0.0679
Anesthesia time (min)	158.5 ± 49.6 [135.3–181.7]	175.5 ± 63.1 [145.9–205.0]	132.7 ± 74.1 [98.0–167.4]	155.6 ± 64.5**†** [138.9–172.2]	0.2134
Total dosage					
Fentanyl (μg kg^–1^)	4.23 ± 1.81 [3.4–5.1]	3.58 ± 1.33 [3.0–4.2]	2.53 ± 1.34* [1.9–3.2]	3.44 ± 1.64 [3.0–3.9]	0.0097
Rrocuronium (mg kg^–1^)	1.12 ± 0.49 [0.9–1.3]	1.28 ± 0.35 [1.1–1.5]	1.46 ± 0.31 [1.3–1.6]	1.29 ± 0.41 [1.2–1.4]	0.0743
Sugammadex (mg kg^–1^) (timing, min before emergence)	2.98 ± 0.73 [2.7–3.3] (−8.8 ± 2.1, [−9.7 to −7.8])	3.09 ± 0.68 [2.8–3.4] (−5.9 ± 2.7 [−7.2 to −4.6])	3.17 ± 1.83 [2.3–4.0] (−6.4 ± 4.8‡ [−8.6 to −4.2)	3.08 ± 1.19 [2.8–3.4] (−7.0 ± 3.6, [−7.9 to −6.1])	0.97720.0603
end of the surgery (min before emergence)	−20.6 ± 4.2 [−22.5 to 18.6]	−16.4 ± 5.1* [−18.7 to −14.0]	−13.6 ± 7.1* [−16.9 to −10.3]	−16.8 ± 6.2 [−18.4 to −15.3]	0.0041

Adult__*SEV*_, adult patients anesthetized with sevoflurane; adult__*DES*_, adult patients anesthetized with desflurane; ped__*SEV*_, pediatric patients anesthetized with sevoflurane. Data are shown as mean ± SD [95% CI]; For group comparisons, one-way analysis of variance (ANOVA) was first used to evaluate variation among group means. Only when the variation among group means was greater than expected by chance (*p* < 0.05), Student-Newman-Keuls multiple comparisons test was performed to evaluate the mean variation between the two groups. *p* < 0.05 was considered significant. *p < 0.05 vs. adult__*SEV*_; ^†^*p* < 0.05 vs. adult__*DES*_; ^‡^*p* < 0.05 vs. adult__*SEV*_ and adult__*DES*_; ^§^ Chi-square test; ^**^not significant between adult__*SEV*_ and adult__*DES*_ groups.

### Poincaré Plot Indices

**FIGURE 1:**
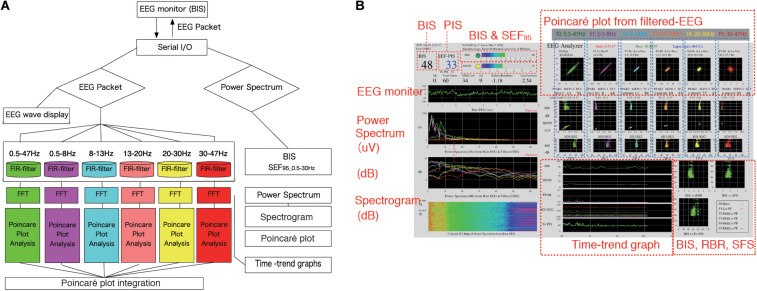
EEG Analyzer software as a real-time monitor of the Poincaré plot. The EEG Analyzer (downloadable for free use from the blog site Science to Medicine, EEG Analyzer ver. 54_GP, http://www.anesth-kpum.org/blog_ts/?p=3169) was developed using the open-source Processing 3.5 software package with the Apache Commons Mathematics Library (version 3.6.1, Apache Software Foundation, Forrest Hill, MD, United States) and a Java Virtual Machine (Oracle, Redwood Shores, CA, United States) with Java class libraries (javax.swing, java.AWT, and java.io packages). **(A)** Flow chart of on-line Poincaré plot analysis of processed EEG data. EEG waves obtained from the BIS VISTA A-3000 were passed through six bandpass filters for fast Fourier transform frequency analysis. Then, processed EEG data at each filtered range was subjected to Poincaré plot analysis during general anesthesia management. **(B)** The EEG Analyzer (connected to a VISTA A-3000 BIS monitor to collect EEG packets through an RS-232 interface) displays BIS, SEF_95_, total power, frequency spectrum, and the Poincaré plot of bandpass-filtered EEG data. Digitized EEG packets with a sampling frequency of 128 Hz were obtained through the serial output of the BIS monitor. Eight seconds of EEG analysis yields 1,024 data points, resulting in a Poincaré plot with 1,024 data points. The Fc-PIS shown on the screen was not yet optimized. BIS, bispectral index; EEG, electroencephalogram; Fc-PIS, frequency analysis-cooperated Poincaré plot-integrated score; SEF_95_, spectral edge frequency below 95% of the power of a given signal is located.

**FIGURE 2:**
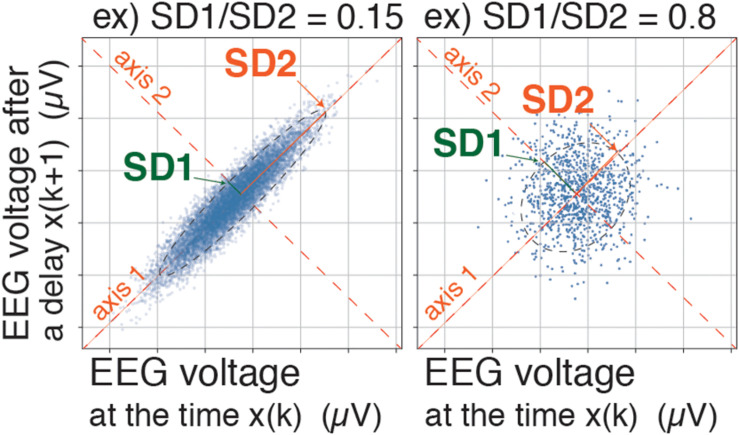
Poincaré plot: SD1/SD2 and Poincaré plot area (SD1 × SD2 × π). A pair of EEG voltages with 1/128-s time lag was plotted in the XY plane, first at a specific time (X: x_(k)_) and then after a time delay (Y: x_(k__+__1)_). SD1 represents the standard deviation of the Poincaré plot perpendicular to the line-of-identity, while SD2 represents the standard deviation of the Poincaré plot along the line-of-identity. EEG, electroencephalogram.

A BIS Quatro sensor was mounted on the frontal region, in accordance with the manufacturer’s recommendations. The digitized EEG packets with a sampling frequency of 128 Hz were obtained through the serial output of the BIS monitor that sent a packet of sixteen sets of EEG μV data (32 bits) and eight packets per second (128 Hz); 8 s of EEG yielded 1,024 data points. Besides, a packet containing processed EEG valuables such as EMGlow (absolute power in the 70–110 Hz range, and, and values in decibel [dB] with respect to 0.0001 μV^2^) was obtained through the serial output of the BIS monitor once every second. For each 8-s data set, raw EEG signals were reconstructed from each digitized EEG packet and processed through six settings of FIR bandpass filters, f0: 0.5–47 Hz, f1: 0.5–8 Hz, f2: 8–13 Hz, f3: 13–20 Hz, f4: 20–30 Hz, and f5: 30–47 Hz (Figure 1A). For each FIR bandpass-filtered EEG dataset, the SEF_95_, TP, and power spectrum were calculated. The Poincaré plot was constructed from the FIR-filtered EEG using 8-s epochs of the EEG signal (1,024 data points). The calculation of Poincaré plot parameters using FIR-filtered EEG A pair of EEG voltages with 1/128-s time lag (the shortest time lag under 128 Hz sampling rate of BIS monitor’s EEG) was plotted in the XY plane, first at a specific time (X: x_(k)_) and then after a time delay (Y: x_(k__+__1)_). To analyze the distribution of EEG patterns in the Poincaré plot, the standard deviation (SD) of the EEG voltage dispersion was measured along and perpendicular to the diagonal line of identity (SD1 and SD2, respectively, Figure 2). SD1 and SD2 represent the minor and the major semi-axes of this fitted ellipse. SD1 is the standard deviation of the distances of points from axis 1 and determines the width of the ellipse (short-term variability), while SD2 equals the standard deviations from axis 2 and length of the ellipse (long-term variability). The equations for the Poincaré plot parameters are as follows (Brennan et al., 2001; Golińska, 2013; Khandoker et al., 2013):

$$S\,D\,1=\frac{1}{\sqrt{2}}\,\text{SD}\,\left(x_{\text{k}}-x_{\text{k}+1}\right)\;$$

$$S\,D\,2=\sqrt{2\,\text{SD}\,{\left(x_{\text{k}}\right)}^{2}-\frac{1}{2}\,S\,D\,{\left(x_{\text{k}}-x_{\text{k}+1}\right)}^{2}}$$

where SD(x*_*k*_*) is a standard deviation of the time series x*_*k*_*.

The SD1/SD2 ratio, which characterizes the sharpness of the scattered pattern, has been reportedly used for estimation of anesthesia depth (Hayashi et al., 2015a,b). However, in the present study, the Poincaré plot area (PP_*A*_) was calculated with the equation SD1 × SD2 × π (Data Sheet 1, Supplementary Method, the example codes of Poincaré plot parameters–calculations with sample data using Python in Jupyter Notebook, and Processing). Our novel approach comprised dividing the PP_*A*_ of each frequency range by the PP_*A*_ of the f0 range (PP_*A_f0*_) and defining the result as the PP_*AR*_ (PP_*AR*_ = PP_*A_fx*_/PP_*A_f0*_, fx = f1∼f5). The value of BIS was simultaneously collected from digital packets received from the BIS monitor, then recorded at 3-s intervals into the output data file.

Prior to this study, we created a software application named EEG Analyzer (downloadable for free use from our blog site, Science to Medicine, EEG Analyzer ver. 54_GP,^1^) (Figure 1B), through which raw EEG signals are obtained from a VISTA A-3000 BIS monitor (Application revision 3.22, Medtronic, Minneapolis, MN, United States) in the legacy mode using an RS-232 interface to a personal computer (Surface Pro 4, Microsoft Co., Redmond, WA, United States). The Processing 3.5 software package (ver. 3.5.3,^2^, MIT Media Lab, Massachusetts Institute of Technology, Cambridge, MA, United States) was used with the Apache Commons Mathematics Library (version 3.6.1,^3^, Apache Software Foundation, Forrest Hill, MD, United States) and a Java Virtual Machine (Oracle, Redwood Shores, CA, United States) with Java class libraries (javax.swing, java.AWT, and java.io packages). Through Processing’s integrated development environment, the program code for the EEG analysis was compiled using a Java Virtual Machine to build an execution file as a standalone application that functions in both Microsoft Windows 10 (Microsoft Co.) and Mac OS X (Apple, Inc. Cupertino, CA, United States).

### Data Processing and Statistics

For parametric regression with curve fitting function and creation of graphs, Microsoft Excel for Mac (ver. 16.16.5, Microsoft Corp., Redmond, WA, United States) and RINEARN Graph 3D (ver. 5.6,^4^, RINEARN, Kyoto, Japan) were used, respectively. Changes in various EEG parameters between two-time points, 10 min before emergence (EM_–__10_) and at the time of emergence (EM_0_), were statistically compared using paired *t*-tests with InStat 3 (GraphPad Software, San Diego, CA, United States). Root mean square error (RMSE) and coefficients of determination (*R*^2^) were calculated as estimators for the regression analyses. For group comparisons of patients’ background characteristics, one-way analysis of variance (ANOVA) was first used to evaluate variation among group means. Only when the variation among group means was greater than expected by chance (*p* < 0.05), Student-Newman-Keuls multiple comparisons test was performed to evaluate the mean variation between the two groups. *p* < 0.05 was considered significant. For time-course comparisons, Student *t*-tests (paired, two tail *p*-value) were used. The *p*-values < 0.05 were considered significant.

## Results

### Time Course of et_*AG*_, BIS, and SEF_95_ From Hypnosis to Emergence

Data were analyzed from 20 adults anesthetized with sevoflurane (adult__*SEV*_ group), 20 adults anesthetized with desflurane (adult__*DES*_ group), and 20 pediatric patients (aged 1–10 years) anesthetized with sevoflurane (ped__*SEV*_ group). All 60 patients from the three groups were also included in a combined group (all__*combined*_ group) (Table 1). All of the patients had the American Society of Anesthesiologists’ physical status values of 1–2 without any neurological diseases (Supplementary Table 1).

**FIGURE 3:**
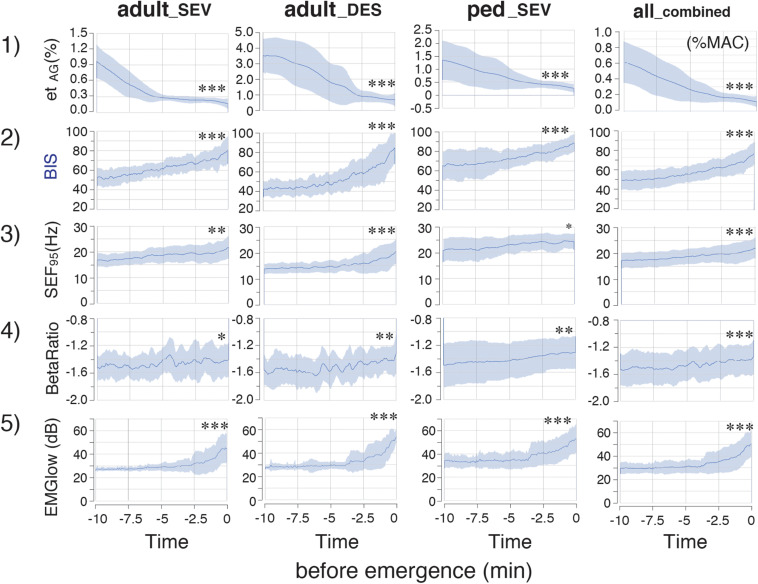
Time-course changes of parameters in the adult__*SEV*_, adult__*DES*_, ped__*SEV*_, and all__*combined*_ groups. et_*AG*_, BIS, SEF_95_ RelativeBetaRatio (BetaRatio), and EMGlow of EEG. Plots of the time course from the hypnotic condition to the awake state of (1) et_*AG*_, (2) BIS, (3) SEF_95_, and the BIS subparameters (4) RelativeBetaRatio (BetaRatio), and (5) EMGlow. adult__*SEV*_, adults anesthetized with sevoflurane (*n* = 20); adult__*DES*_, adults anesthetized with desflurane (*n* = 20); ped__*SEV*_, pediatric patients anesthetized with sevoflurane (*n* = 20); all__*combined*_, all patients from the three groups (*n* = 60). Data are shown as mean ± SD. ^∗^*p* < 0.05, ^∗∗^*p* < 0.01, and ^∗∗∗^*p* < 0.001 by Student’s paired *t*-test between the values at EM_–__10_ and EM_0_. BIS, bispectral index; EM_–__10_, 10 min before emergence; EM_0_, at the time of emergence; et_*AG*_, end-tidal anesthetic gas concentration; SEF_95_, spectral edge frequency below which 95% of the power of a given signal is located; EMGlow, a BIS monitor-derived electromyography parameter corresponding to absolute power in the 70–110 Hz range, and values in decibel (dB) with respect to 0.0001 μV^2^.

In the time-course from the hypnotic state to arousal during the 10-min process of emergence from GA (Figure 3), et_*SEV*_ in the adult__*SEV*_ group, et_*DES*_ in the adult__*DES*_ group, and et_*SEV*_ in the ped__*SEV*_ group decreased from 0.97 ± 0.32, 3.71 ± 0.84, and 1.34 ± 0.75 to 0.17 ± 0.10, 0.68 ± 0.42, and 0.27 ± 0.16, respectively (*p <* 0.05 for all three groups). BIS values of the three groups increased from 52.9 ± 8.9, 41.9 ± 8.8, and 65.4 ± 14.9 to 80.5 ± 13.8, 84.7 ± 16.4, and 88.4 ± 9.1, respectively (*p <* 0.05 for all three groups). SEF_95_ increased from 16.8 ± 2.5 Hz, 13.6 ± 1.9 Hz, and 20.8 ± 4.5 Hz to 21.7 ± 4.4 Hz, 20.3 ± 4.6 Hz, and 24.1 ± 3.3 Hz, respectively (*p <* 0.05 for all three groups). RelativeBetaRatio (RBR), a subparameter of BIS, tended to increase (*p <* 0.05). EMGlow, which corresponds to the EMG power of 70–110 Hz, also increased for all three groups (*p <* 0.05). In the all__*combined*_ group, significant differences (*p <* 0.05) between EM_–__10_ and EM_0_ were detected for et_*AG*_, BIS, SEF_95_, RBR, and EMGlow (Figure 3). Supplementary Tables 2, 3 show the detailed measurements in each of the four groups.

### Time Course of TP and Poincaré Plot Parameters From Hypnosis to Arousal

The time-course changes of TP were analyzed at each frequency band. In each frequency band, TP decreased over time, although small increases were observed in the last few minutes of TP_*f5*_ in the adult__*SEV*_ group, as well as in the last few minutes of TP_*f4*_ and TP_*f5*_ in the adult__*DES*_ group (*p <* 0.05) (Supplementary Figure 1A). Next, Poincaré plot parameters (SD1 and SD2), which were obtained from the Poincaré plot-analysis of a pair of FIR-filtered EEG μV with a 1/128-s time lag, were analyzed at each frequency band. The time-course changes of SD1/SD2 depended strongly on the frequency band: gradual increases of SD1/SD2 were observed in f0, f3, f4, and f5, while gradual decreases were observed in f1 and f2 (Supplementary Figure 1B). However, the overall mean value changes of SD1/SD2 across all frequency bands were small compared to their standard deviations.

**FIGURE 4:**
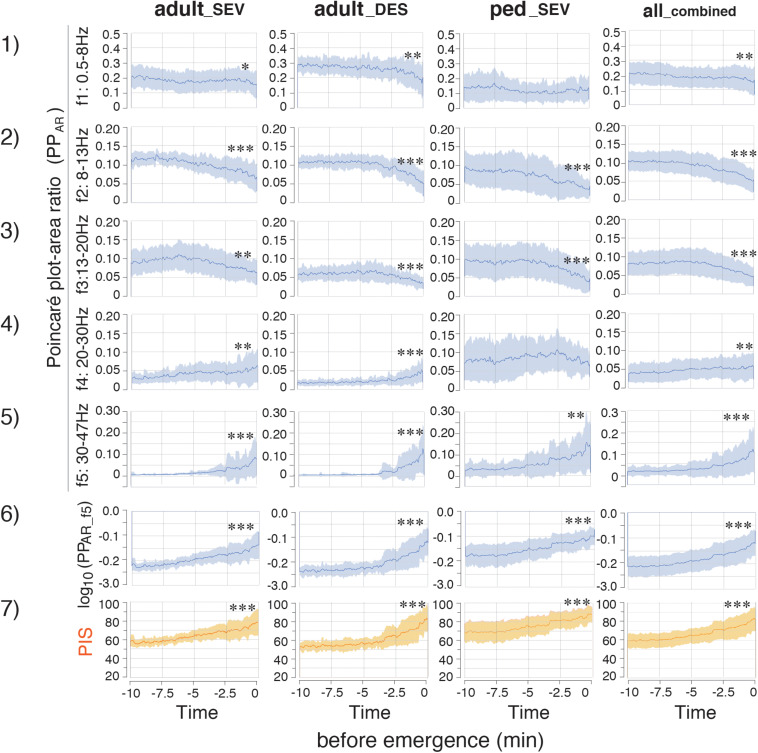
Time-course changes of parameters in the adult__*SEV*_, adult__*DES*_, ped__*SEV*_, and all__*combined*_ groups. (1–5) Plots of the time course of the PP_*AR*_ (Poincaré plot area of each frequency-range/Poincaré plot area of the f0: 0.5–47 Hz range) throughout the time course from the hypnotic condition to the awake state. (6) log_10_(PP_*AR_f5*_) and PIS. (7) Poincaré plot-integrated score (PIS). PIS = 25 × log_10_(PP_*AR_f5*_) + 112.5. adult__*SEV*_, adults anesthetized with sevoflurane (*n* = 20); adult__*DES*_, adults anesthetized with desflurane (*n* = 20); ped__*SEV*_, pediatric patients anesthetized with sevoflurane (*n* = 20); all__*combined*_ group, all patients from the three groups (*n* = 60). Data are shown as mean ± SD. ^∗^*p* < 0.05, ^∗∗^*p* < 0.01, and ^∗∗∗^*p* < 0.001 by Student’s paired *t*-test between the values at EM_–__10_ and EM_0_. EM_–__10_, 10 min before emergence; EM_0_, at the time of emergence; PP_*AR*_, Poincaré plot-area ratio.

**FIGURE 5:**
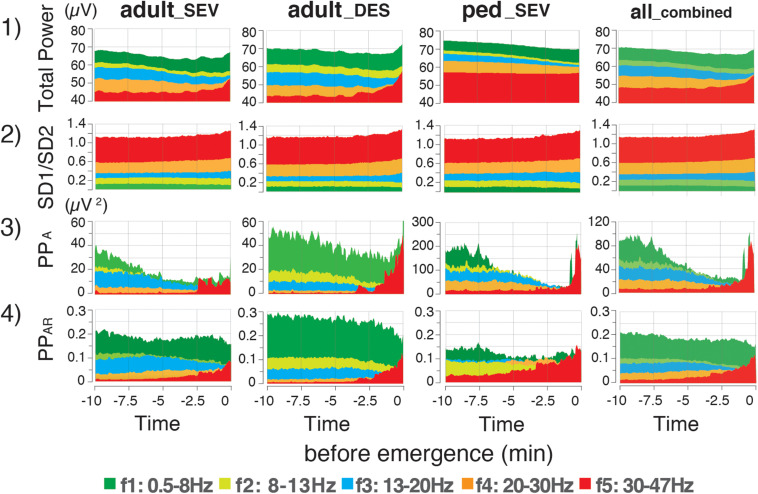
Time-course changes of mean values of EEG parameters in the adult__*SEV*_, adult__*DES*_, and ped__*SEV*_ groups, and etAG in the all__*combined*_ group. Mean values of (1) total power, (2) SD1/SD2 of the Poincaré plot, (3) Poincaré plot area (PP_*A*_), and (4) Poincaré plot-area ratio (PP_*AR*_: PP_*A*_ of each frequency-range/PP_*A*_ value of the f0:0.5–47 Hz range) throughout the time course from the hypnotic condition to the awake state. Data are shown as means. adult__*SEV*_, adults anesthetized with sevoflurane (*n* = 20); adult__*DES*_, adults anesthetized with desflurane (*n* = 20); ped__*SEV*_, pediatric patients anesthetized with sevoflurane (*n* = 20); all__*combined*_ group, all patients from the three groups (*n* = 60). Plots of the time course range from the hypnotic condition (EM_–__10_) to the awake state (EM_0_). EM_–__10_, 10 min before emergence; EM_0_, at the time of emergence; PP_*AR*_, Poincaré plot-area ratio.

PP_*A*_, which is calculated from SD2 × SD2 × π, changed dynamically during the emergence process (Supplementary Figure 1C). The absolute values of PP_*A*_ in the ped__*SEV*_ group were approximately five times larger than those values in the adult groups. The changes in PP_*A*_ were not consistent over time: the values of PP_*A_f4*_ and PP_*A_f5*_ suddenly increased over the last 2–3 min in all three groups (*p <* 0.05, PP_*A_f5*_). A similar increase in PP_*A_f0*_ was observed during the final phase of emergence. Next, the calculated absolute values of PP_*A*_ were significantly different among the three groups. Therefore, the adjustment was performed: the ratios of PP_*A*_ at each frequency range to PP_*A_f0*_ were calculated as PP_*AR*_ (PP_*AR*_ of each frequency-range/PP_*AR*_ of the f0: 0.5–47 Hz range) throughout the time course from the hypnotic condition to the awake state. The results showed that the PP_*AR*_ values for all three groups fit into the same plotting range. Drastic changes in PP_*AR*_ were detected among f1–f5 across all three groups (Figure 4). PP_*AR_f2*_ and PP_*AR_f3*_ significantly decreased (*p <* 0.05 for all three groups), while PP_*AR_f5*_ significantly increased (*p <* 0.01 for all three groups). The time-course changes of PP_*AR_f5*_ demonstrated notable changes 2–3 min before emergence: although the values of PP_*AR_f5*_ remained near 0 for the first 7–8 min, the values began to increase in the final few minutes before emergence. The mean changes in TP, SD1/SD2, PP_*A*_, and PP_*AR*_ values in each frequency range over time are summarized in Figure 5. Changes of parameters in f5, such as PP_*A_f5*_ and PP_*AR_f5*_, were remarkable among all five hierarchical frequency bands. In the all__*combined*_ group, for et_*AG*_, significant differences (*p <* 0.05) were detected in both SD1/SD2 and PP_*AR*_ at all five hierarchical frequency bands (Figure 4 and Supplementary Table 2B).

### Correlations of SD1/SD2 With et_*AG*_ and BIS, and Correlations of PP_*AR*_ With et_*AG*_ and BIS

**FIGURE 6:**
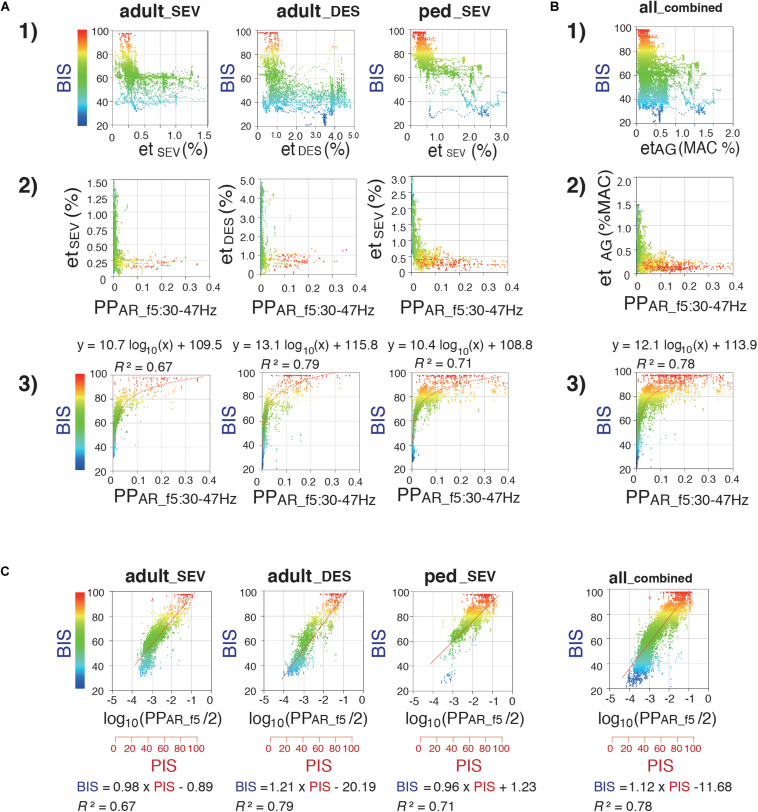
Relationship between Poincaré plot-area ratio at f5:30–47 Hz (PP_*AR_f5*_), BIS, and et_*AG*_. Scatter plots display correlations between (1) et_*AG*_ (%) and BIS, (2) PP_*AR_f5*_ and et_*AG*_ (%), and (3) PP_*AR_f5*_ and BIS **(A)** in the adult__*SEV*_, adult__*DES*_, and ped__*SEV*_ groups and **(B)** in the all__*combined*_ group. (4) 3D-scatter plot between end-tidal concentrations (%MAC), PP_*AR_f5*_, and BIS in the all__*combined*_ group. **(C)** Semi-logarithmic scatter plots between Poincaré plot-integrated score (PIS) and BIS in the adult__*SEV*_, adult__*DES*_, ped__*SEV*_, and all__*combined*_ groups. PIS = 25 × log_10_(PP_*AR_f5*_)+112.5. Minimum alveolar concentration at 50% (MAC) values of sevoflurane and desflurane were calculated by using 1.71 and 7.25, respectively. adult__*SEV*_, adult patients anesthetized with sevoflurane (*n* = 20); adult__*DES*_, adult patients anesthetized with desflurane (*n* = 20); ped__*SEV*_, pediatric patients anesthetized with sevoflurane (*n* = 20); all__*combined*_, all patients from the three groups (*n* = 60). RINEARN Graph 3D (free software ver 5.6, https://www.rinearn.com, RINEARN, Kyoto, Japan) was used for the creation of graphs. BIS, bispectral index; et_*AG*_, end-tidal anesthetic gas; et_*DES*_, end-tidal sevoflurane concentration; et_*SEV*_, end-tidal sevoflurane concentration; PIS, Poincaré plot-integrated score; PP_*AR_f5*_, Poincaré plot-area ratio at f5:30–47 Hz; *R*^2^, coefficient of determination.

Next, using 4,000 data points obtained from 20 patients, the relationships of SD1/SD2 with et_*AG*_ and BIS were evaluated (Supplementary Figure 2A). Differences in the distributions of SD1/SD2, compared with et_*AG*_ and BIS, are present in each frequency range plot. Analysis of the relationship between SD1/SD2 and et_*AG*_ showed that when et_*AG*_ decreased, SD1/SD2 tended to be dispersed across all plots. Overall, both linear and logarithmic correlations between SD1/SD2 of six frequency bands and BIS in all three groups were low (*R*^2^ ≤ 0.5). The relationships of PP_*AR*_ with et_*AG*_ and BIS were also evaluated (Supplementary Figure 2B). Scatter plots were created to assess PP_*AR*_ at five frequency bands, compared with et_*AG*_. In plots between PP_*AR_f5*_ and et_*AG*_, hyperbolic shapes with large variation at low et_*AG*_ values were observed in all three groups. Regarding the linear correlation between PP_*AR_f1*_ and BIS, the *R*^2^ values in all three groups were less than 0.44. PP_*AR_f2*_ and PP_*AR_f3*_ showed biphasic changes against BIS in all three groups. Logarithmic correlations between PP_*AR_f4*_ and BIS showed *R*^2^ values of 0.36, 0.59, and 0.27 for adult__*SEV*_, adult__*DES*_, and ped__*SEV*_ groups. The relationships between PP_*AR_f5*_ and BIS in the three groups are summarized in Figure 6. Regarding the logarithmic correlations between PP_*AR_f5*_ and BIS, *R*^2^ values were 0.67, 0.79, and 0.71, respectively [Figure 6A(3)]. Regarding the logarithmic correlation between PP_*AR_f5*_ and BIS in the all__*combined*_ group, *R*^2^ was 0.78 [Figure 6B(3)].

### Correlations of log_10_(PP_*AR_f5*_) With BIS

During the emergence process, the all__*combined*_ group exhibited a gradual reduction of the average et_*AG*_ from 0.61 ± 0.30% to 0.12 ± 0.08%; furthermore, the average BIS gradually increased from 53.4 ± 14.7 to 84.6 ± 13.6 [Figure 3(1, 2)]. Because the relationship between PP_*AR_f5*_ and BIS fit well to a logarithmic curve with high *R*^2^ values in all three groups, the relationship between log_10_(PP_*AR_f5*_) and BIS was fitted using linear regression (Figure 6C). Thus, the depth of anesthesia captured by the logarithmic value of PP_*AR_f5*_, or by BIS, changed in a nearly linear manner from the state of anesthesia to emergence. Therefore, BIS is an index that can be easily understood by anesthesiologists because it converts the depth of anesthesia into a score ranging from 0 to 100; the logarithmic value of PP_*AR_f5*_ can also be easily converted to a 0–100 range. From the data of our 60 cases, PP_*AR_f5*_ = 0.3162 (logarithmic value −0.5) was defined as Poincaré plot-integrated score (PIS) = 100, and PP_*AR_f5*_ = 0.0031623 (logarithmic value −2.5) was defined as PIS = 50. The linear regression conversion formula for calculating the PIS value from PP_*AR_f5*_ then became PIS = 25 × log_10_ (PP_*AR_f5*_)+112.5. Figure 4(6, 7) show the time-course changes of log_10_(PP_*AR_f5*_) and calculated PIS values in the four groups. *R*^2^ between PIS and BIS in the three groups, which was between 0.67 and 0.79 (Figure 6C), is mathematically identical to the *R*^2^ between the original PP_*AR_f5*_ and BIS [Figure 6A(3)]. In the all__*combined*_ group, the regression equation BIS = 1.12 × PIS −11.68 was obtained (*R*^2^ = 0.78) (Figure 6C).

### Correlations of EMGlow With BIS and PP_*AR_f5*_

**FIGURE 7:**
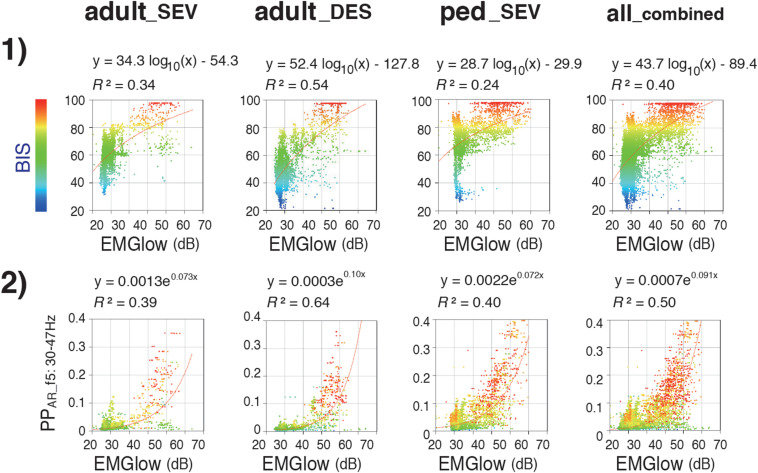
Relationships of EMGlow with BIS and Poincaré plot parameter PP_*AR_f5*_. Scatter plots display correlations between (1) EMGlow and BIS and (2) EMGlow and PP_*AR_f5*_ in the adult__*SEV*_, adult__*DES*_, and ped__*SEV*_ groups and in the all__*combined*_ group. adult__*SEV*_, adult patients anesthetized with sevoflurane (*n* = 20); adult__*DES*_, adult patients anesthetized with desflurane (*n* = 20); ped__*SEV*_, pediatric patients anesthetized with sevoflurane (*n* = 20); all__*combined*_, all patients from the three groups (*n* = 60). RINEARN Graph 3D (free software ver 5.6, https://www.rinearn.com, RINEARN, Kyoto, Japan) was used for the creation of graphs. EMGlow, a BIS monitor-derived electromyography parameter corresponding to absolute power in the 70–110 Hz range, and values in decibel (dB) with respect to 0.0001 μV^2^; BIS, bispectral index; PP_*AR_f5*_, Poincaré plot-area ratio at f5:30–47 Hz; *R*^2^, coefficient of determination.

While, during the emergence process, the all__*combined*_ group exhibited a gradual increase of the average BIS from 53.4 ± 14.7 to 84.6 ± 13.6 [Figure 3(2)], the average EMGlow gradually increased from 2943.5 ± 465.7 to 5063.9 ± 1082.0 (Figure 3(5), Supplementary Table 2B). *R*^2^ of the logarithmic regressions between EMGlow and BIS in the three groups were between 0.24 and 0.54 [Figure 7(1)], and *R*^2^ of the exponential regressions between EMGlow and PP_*AR_f5*_ were between 0.39 and 0.64 [Figure 7(2)]. EMGlow is an absolute power at the range of 70–110 Hz. These results implied that, although the *R*^2^ of the regressions between EMGlow and PP_*AR_f5*_ was not so great as that between BIS and PP_*AR_f5*_, a part of EMG activity at the gamma range of 30–47 Hz probably influenced the calculations of BIS and PP_*AR*_____*f5*_ with a non-negligible level.

### Case Examples: Video Clips of the Poincaré Plot, EEG Spectrum, and Spectrogram

**FIGURE 8:**
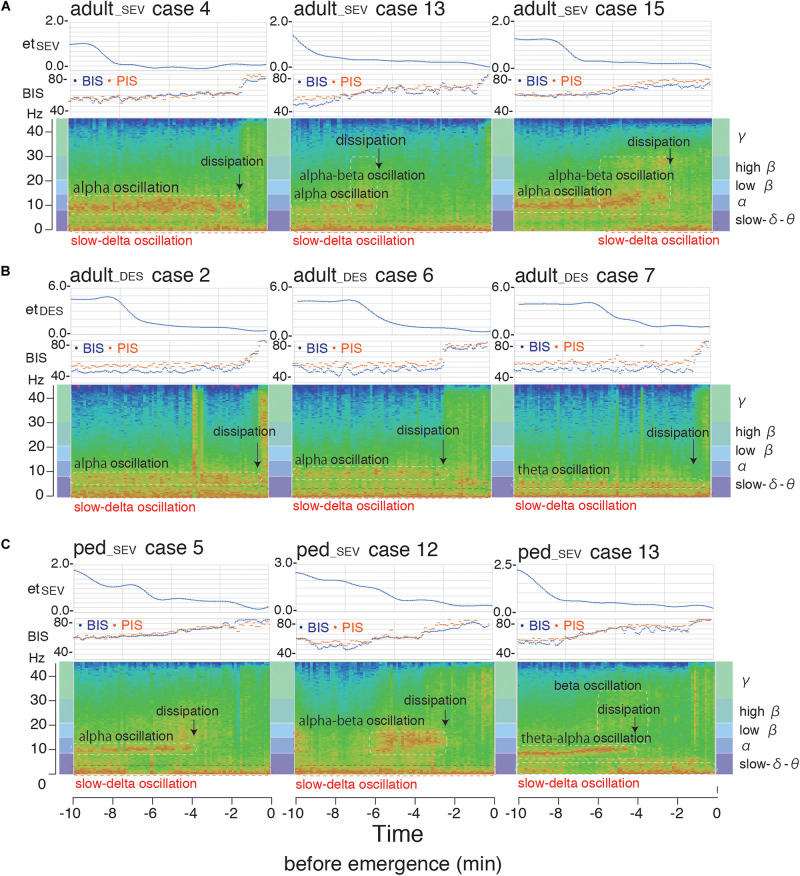
Spectrogram from the hypnotic state to emergence from general anesthesia. Spectrograms of three representative cases [**(A)** adult__*SEV*_, **(B)** adult__*DES*_, and **(C)** ped__*SEV*_] from each of the three patient groups with time trends of BIS, PIS, et_*SEV*_, and et_*DES*_ at 10 min before emergence from general anesthesia. Theta-to-alpha oscillations were observed before the dissipation points, after which beta-to-gamma oscillations were observed with abrupt increases of BIS and PIS. adult__*SEV*_, adult patients anesthetized with sevoflurane; adult__*DES*_, adult patients anesthetized with desflurane; ped__*SEV*_, pediatric patients anesthetized with sevoflurane; BIS, bispectral index; et_*SEV*_, end-tidal sevoflurane concentration; et_*DES*_, end-tidal sevoflurane concentration; PIS, Poincaré plot-integrated score.

In the case-by-case plots of BIS and PIS (Supplementary Figure 3), the overall time-course change of calculated PIS fit well to the change of measured BIS in each case, despite slight differences in correlations detected by RMSE and *R*^2^ among the three groups (Supplementary Table 3). One representative case from each group of the three patient groups (A: adult__*SEV*_, B: adult__*DES*_, and C: ped__*SEV*_) is shown as a movie clip (Supplementary Video Clips) with spectrograms, time trends of BIS, PIS, and et_*SEV*_ or et_*DES*_ at 10 min before emergence from GA. Corresponding spectrograms of the above three cases from each group are shown with the corresponding time trends of BIS, PIS, et_*SEV*_, or et_*DES*_ (Figure 8). Theta-to-alpha oscillations were observed before the dissipation points, followed by the emergence of beta-to-gamma oscillations with abrupt increases in BIS and PIS.

## Discussion

Electroencephalography signals represent the total accumulation of action potentials of each nerve cell in the cerebral cortex. A frequency-dependent method is generally used to reduce the complexity of those data. The Poincaré plot is another mathematical approach for the evaluation of those complex data. There is no difference in the neurobiological basis that serves as the starting point. This study developed an EEG analyzer that can display multiple aspects of EEG signals, including unprocessed EEG waves, power spectra, spectrograms, and the Poincaré plot with accompanying parameters, on a single screen. As a result, the PP_*A*_ of the Poincaré plot reflected the factors of both EEG power and complexity; therefore, PP_*A*_ changed dynamically during the process of emergence from GA. The PP_*AR*_ indicates the power and complexity of a specific frequency range of EEG, relative to those attributes of the full EEG frequency range. Notably, regardless of the anesthetic type and patient age, PP_*A*__*R*___f5_ uniformly increased in the last few minutes before emergence across all three groups. The logarithmic regression results indicated that PP_*AR_f5*_ exhibited positive correlations with BIS, with high *R*^2^ values. These results suggested that, during anesthesia depth change, EEG signals contain more high-frequency gamma-wave components and become more complex with time; these tendencies were reflected in the trend of PP_*AR_f5*_, although we acknowledge an undefined theoretical foundation in the Poincaré plot analysis for processed EEG.

Because the logarithmic regression analysis between PP_*AR_f5*_ and BIS showed a positive correlation with BIS, with high *R*^2^ values, the logarithmic value of PP_*AR_f5*_ changed linearly, similar to the linear increase of BIS during the investigated 10-min period. Thus, for the period in which the change in depth of anesthesia is difficult to perceive, as the patient does not exhibit active behavior, the logarithm of PP_*AR_f5*_ could serve as a simple, independent non-proprietary indicator of GA depth. There were slight differences in regression model error between adult and pediatric patients, as well as between sevoflurane and desflurane. Many recent reports have indicated the need for new monitoring algorithms or calibration to better adjust for age-specific EEG characteristics in both pediatric (Fuentes et al., 2008; Sciusco et al., 2017; Beekoo et al., 2019; Rigouzzo et al., 2019; Wang et al., 2019) and older patients (Purdon P. et al., 2015; Ni et al., 2019; Kreuzer et al., 2020). It may be necessary to incorporate these regulatory mechanisms to obtain better correlations with anesthesia depth.

Gamma-band EEG oscillations above 40 Hz have been recognized as a critical marker of the conscious state (Desmedt and Tomberg, 1994; Franken et al., 1994; Menon et al., 1996; Gross and Gotman, 1999). The BIS proprietary algorithm includes subparameters such as burst suppression ratio, RBR, and sync fast slow (SFS), with multiple regression equations (Sigl and Chamoun, 1994; Rampil, 1998). RBR is defined as the logarithmic ratio of gamma-range spectral power (*P*_30__–__47 *Hz*_) to the spectral power of the 11–20 Hz frequency band (*P*_11__–__20 *Hz*_). Several studies have indicated that BIS values are highly dependent on RBR when the value of BIS is greater than 60 (Morimoto et al., 2004; Lee et al., 2019). SFS is the logarithm of the ratio of the bispectral power in the 40–47 Hz waveband to the bispectral power in the 0.5–47 Hz band (Rampil, 1998). At surgical levels of isoflurane anesthesia, BIS and SFS are closely correlated, as are BIS and SEF_95_ (Morimoto et al., 2004). A good correlation between BIS, SEF_95_, and SD1/SD2 of the Poincaré plot was also reported at surgical levels of sevoflurane inhalational anesthesia (Hayashi et al., 2015a). However, these strong correlations among BIS, SEF_95_, and SD1/SD2 of the Poincaré plot have probably been only observed at the surgical level of anesthesia, but not at the shallow anesthetic level during the arousal phase.

Previous reports mentioned that increased BIS was related to an EMG signal associated with muscle relaxation in the awake state (Bruhn et al., 2000a; Sleigh et al., 2001; Messner et al., 2003; Schuller et al., 2015), or reversal of muscle relaxation by sugammadex in the hypnotic state (Aho et al., 2012). Therefore, EMG power may be suitable as an index of the BIS range. Including the non-negligible influence of frontal EMG on the high-frequency band of frontal EEG, the BIS values generally reflect changes in gamma-frequency EEG components, which probably contaminates with EMG at the level that cannot be ignored. In addition to BIS, the spectral entropy monitor uses EEG irregularity and computes state entropy over the frequency range of 0.8–32 Hz, reflecting the EEG-dominant part of the spectrum (Ellerkmann et al., 2004). Additionally, response entropy was computed over the frequency band of 0.8–47 Hz, including both the EEG- and EMG-dominant parts of the recorded spectrum Ellerkmann et al., 2004). Our main result—PP_*A_f5*_ or logarithmic PP_*A_f5*_ had the best correlation with BIS during the emergence process—seems reasonable, considering that both BIS and spectral entropy use measurements of gamma-frequency EEG as critical components of subparameters that represent anesthetic depth. Although one report demonstrated that the average change in EMG signal was approximately one-tenth of the magnitude of the EEG signal when the submental EMG signal was used to estimate the frontal EMG signal (Sleigh et al., 2001), it is necessary to understand that EEG in the gamma region utilized by BIS and our PP_*AR_f5*_ are highly affected by the non-negligible power of EMG.

A limitation of this study was that it focused solely on the emergence process from GA; the study did not include a parametric analysis involving patients in a more profound state of GA. Because the BIS uses at least three subparameters of anesthesia state, the combination of SEF_95_ with the PP_*AR*_ could be used to create a new depth-of-anesthesia monitor with a known algorithm. We acknowledge that the finding associated with PP_*AR_f5*_ may only be applicable for anesthetic phases ranging between light anesthesia level and awareness. In this study, poor correlations were observed between etAG and anesthetic depth parameters, such as BIS and PP_*AR_f5*_. Because we used a semi-closed circuit for GA, measured end-tidal anesthetic concentration would not reflect the “true” concentration. Pharmacokinetics simulation of putative brain concentration of anesthetic during emergence from anesthesia will sure lead to more appropriate assessment of anesthetic action on EEG changes, although we could not handle it for technical reasons in this study. The influence of EMG from the forehead also requires further examination through case-control studies because the effects of EMG on BIS have been evaluated in awake volunteers (Messner et al., 2003; Schuller et al., 2015). The use of standard parameters derived from a non-proprietary algorithm to indicate anesthetic depth could clarify outcomes of clinical trials that have targeted prevention of awareness or delirium by using different parameters derived from specific proprietary algorithms (Abbott and Pearse, 2019; Whitlock and Avidan, 2020). Another limitation of this study involved its observational design; thus, we could not eliminate the influences of noxious stimuli from surgical procedures near the end of surgery. Variation in opioid (fentanyl) dosage may also have affected patient awareness. Further analyses (e.g., controlled trials) could enhance the clinical utility of evaluating the PP_*AR*_ as an indicator of anesthetic depth. Ultimately, we aim to propose an open protocol for an anesthetic depth algorithm that can be used by anesthesiologists.

The clinical use of EEG-based indices for anesthetic depth monitoring remains controversial due to the lack of a neurophysiological interaction between drug-specific neurophysiological signatures and the meaning of the indices, as noted in a recent educational review (Purdon P.L. et al., 2015). We currently agree with the importance of evaluation of unprocessed EEG data; spectrograms have received greater emphasis in terms of the interpretation of anesthetic-induced brain states defined by drug-specific neurophysiological signatures. Furthermore, the relationship between age and postoperative cognitive impairment is becoming an important consideration for measuring anesthesia depth (Purdon P. et al., 2015; Ni et al., 2019; Kreuzer et al., 2020). Among the various components of EEG-guided anesthesia, our results showed an important aspect, such that Poincaré plot parameters obtained from EEG signals processed through gamma-band frequency filters revealed a notable change during emergence from GA, although it cannot be denied the influence of EMG power in this EEG range. In conclusion, our results suggest that under inhalational GA, the logarithm of PP_*AR_f5*_ (PIS) could serve as a non-proprietary anesthetic depth parameter independent of the proprietary BIS algorithm.

## Data Availability Statement

The original contributions presented in the study are included in the article/Supplementary Material, further inquiries can be directed to the corresponding author/s.

## Ethics Statement

The studies involving human participants were reviewed and approved by The Institutional Review Board for Human Experiments at the Kyoto Prefectural University of Medicine. Written informed consent from the participants’ legal guardian/next of kin was not required to participate in this study in accordance with the national legislation and the institutional requirements.

## Author Contributions

KH was the first author and performed the data collection and data analysis. AK, KA, MK, and MS performed manuscript preparation. TS was the principal investigator responsible for study conception and design, data analysis, and manuscript preparation. All authors contributed to the article and approved the submitted version.

## Conflict of Interest

The authors declare that the research was conducted in the absence of any commercial or financial relationships that could be construed as a potential conflict of interest.

## Footnotes

1 http://www.anesth-kpum.org/blog_ts/?p=3169

2 https://processing.org

3 https://commons.apache.org

4 https://www.rinearn.com

## Supplementary Material

Supplementary Figure 1 Total EEG power and Poincaré plot parameters throughout the time course from the hypnotic condition to the awake state in anesthetized patients. **(A)** Total preprocessed EEG power after passing through one of five bandpass filters, **(B)** SD1/SD2 of the Poincaré plot of the preprocessed EEG signal after passing through one of six bandpass filters, **(C)** Area of the Poincaré plot (PP_*A*_) of the processed EEG signal after passing through one of six bandpass filters.

Supplementary Figure 2 Relationships of Poincaré plot parameters with BIS and etAG. **(A)** The relationships of Poincaré plot SD1/SD2 with BIS and etAG. **(B)** The relationships of Poincaré plot Poincaré plot area ratio (PP_*AR*_) with BIS and etAG.

Supplementary Figure 3 Case-by-case plots of time-course change of PIS and BIS, and linear regressions between PIS and BIS. **(A)** Adult sevoflurane (adult__*S**E**V*_) group. **(B)** Adult sevoflurane (adult__*D**E**S*_) group. **(C)** Adult sevoflurane (ped__*S**E**V*_) group.

Supplementary Table 1 Detailed information about patients and anesthesia management.

Supplementary Table 2 Comparison of changes in EEG parameters. **(A)** The comparison between three groups. **(B)** The comparison in the all__*c**o**m**b**i**n**e**d*_ group.

Supplementary Table 3 Evaluation of linear regression of the three groups.

Supplementary Data Sheet 1 The example codes of Poincaré plot parameters – calculations with sample data using Python in Jupyter Notebook, and Processing.

Supplementary Video Clips (Separate files, three clips) one case from each group, 10 min movie clips: Three representative cases from each group are shown as movie clips (35–36 s), which were displayed by decoding from the recorded binary files under “REVIEW mode” of the EEG analyzer and illustrate a 10 min period from the hypnotic state to arousal. The movie clips include the screen of the EEG analyzer (ver. 49_SR), including the Poincaré plot of processed EEG through specific bandpass filters and the EEG power spectrum. (Please ignore the Fc-PIS values on the screen because the algorithm for the calculation of Fc-PIS value was not optimized when we recorded the analyses. Fc-PIS: frequency analysis-cooperated Poincaré plot-integrated score).

Supplementary Video 1 Supplementary_Video_1_adult_SEv_case4_ EEGanalyzer.mp4, EEG analyzer screen (10 min) of adult_SEV case #4.

Supplementary Video 2 Supplementary_Video_2_adult_DES_ case2_EEGanalyzer.mp4, EEG analyzer screen (10 min) of adult_DES case #2.

Supplementary Video 3 Supplementary_Video_3_ped_SEV_case5_ EEGanalyzer.mp4, EEG analyzer screen (10 min) of ped_SEV case #5.

## References

[B1] Abbott T. E. F. Pearse R. M. (2019). Depth of anesthesia and postoperative delirium. *JAMA* 321 459–460. 10.1001/jama.2019.0164 30721279

[B2] Aho A. J. Kamata K. Yli-Hankala A. Lyytikäinen L. P. Kulkas A. Jäntti V. (2012). Elevated BIS and entropy values after sugammadex or neostigmine: an electroencephalographic or electromyographic phenomenon? *Acta Anaesthesiol. Scand.* 56 465–473. 10.1111/j.1399-6576.2011.02647.x 22289106

[B3] Avidan M. S. Zhang L. Burnside B. A. Finkel K. J. Searleman A. C. Selvidge J. A. (2008). Anesthesia awareness and the bispectral index. *N. Engl. J. Med.* 358 1097–1108. 10.1056/NEJMoa0707361 18337600

[B4] Avidan M. S. Jacobsohn E. Glick D. Burnside B. A. Zhang L. Villafranca A. (2011). Prevention of intraoperative awareness in a high-risk surgical population. *N. Engl. J Med.* 365 591–600. 10.1056/NEJMoa1100403 21848460

[B5] Becker K. Schneider G. Eder M. Ranft A. Kochs E. F. Zieglgänsberger W. (2010). Anaesthesia monitoring by recurrence quantification analysis of EEG data. *PLoS One* 5:e8876. 10.1371/journal.pone.0008876 20126649 PMC2811188

[B6] Beekoo D. Yuan K. Dai S. Chen L. Di M. Wang S. (2019). Analyzing electroencephalography (EEG) waves provides a reliable tool to assess the depth of sevoflurane anesthesia in pediatric patients. *Med. Sci. Monit.* 25 4035–4040. 10.12659/MSM.915640 31146277 PMC6559006

[B7] Bischoff P. Rundshagen I. (2011). Awareness under general anesthesia. *Dtsch Arztebl. Int.* 108 1–7. 10.3238/arztebl.2011.0001 21285993 PMC3026393

[B8] Brennan M. Palaniswami M. Kamen P. (2001). Do existing measures of Poincaré plot geometry reflect nonlinear features of heart rate variability. *IEEE Trans. Biomed. Eg.* 48 1342–1347. 10.1109/10.9593304 11686633

[B9] Bruhn J. Bouillon T. W. Shafer S. L. (2000a). Electromyographic activity falsely elevates the bispectral index. *Anesthesiology* 92 1485–1487. 10.1097/00000542-200005000-200005042 10781298

[B10] Bruhn J. Röpcke H. Hoeft A. (2000b). Approximate entropy as an electroencephalographic measure of anesthetic drug effect during desflurane anesthesia. *Anesthesiology* 92 715–726. 10.1097/00000542-200003000-00016 10719951

[B11] Carrasco S. Gaitan M. J. Gonzalez R. Yanez O. (2001). Correlation among Poincaré plot indexes and time and frequency domain measures of heart rate variability. *J. Med. Eng. Technol.* 25 240–248. 10.1080/03091900110086651 11780765

[B12] Chan M. T. Cheng B. C. Lee T. M. Gin T. Coda Trial and Group. (2013). BIS-guided anesthesia decreases postoperative delirium and cognitive decline. *J. Neurosurg. Anesthesiol.* 25 33–42. 10.1097/ANA.0b013e3182712fba 23027226

[B13] Chang C. Raven E. P. Duyn J. H. (2016). Brain-heart interactions: challenges and opportunities with functional magnetic resonance imaging at ultra-high field. *Philos. Trans. A Math. Phys. Eng. Sci.* 374:20150188. 10.1098/rsta.2015.0188 27044994 PMC4822447

[B14] Cole D. J. Kharasch E. D. (2018). Postoperative brain function: toward a better understanding and the American Society of Anesthesiologists perioperative brain health initiative. *Anesthesiology* 129 861–863. 10.1097/ALN.0000000000002085 30325803

[B15] Desmedt J. E. Tomberg C. (1994). Transient phase-locking of 40 Hz electrical oscillations in prefrontal and parietal human cortex reflects the process of conscious somatic perception. *Neurosci. Lett.* 168 126–129. 10.1016/0304-3940(94)90432-4 8028764

[B16] Ekman A. Lindholm M. L. Lennmarken C. Sandin R. (2004). Reduction in the incidence of awareness using BIS monitoring. *Acta Anaesthesiol. Scand.* 48 20–26. 10.1111/j.1399-6576.2004.00260.x 14674969

[B17] Ellerkmann R. K. Liermann V. M. Alves T. M. Wenningmann I. Kreuer S. Wilhelm W. (2004). Spectral entropy and bispectral index as measures of the electroencephalographic effects of sevoflurane. *Anesthesiology* 101 1275–1282. 10.1097/00000542-200412000-00006 15564933

[B18] Fahy B. G. Chau D. F. (2018). The technology of processed electroencephalogram monitoring devices for assessment of depth of anesthesia. *Anesth. Analg.* 126 111–117. 10.1213/ANE.0000000000002331 28786839

[B19] Franken P. Dijk D. J. Toblerm I. Borbely A. A. (1994). High-frequency components of the rat electrocorticogram are modulated by the vigilance states. *Neurosci. Lett.* 167 89–92. 10.1016/0304-3940(94)91034-0 8177536

[B20] Fuentes R. Cortinez L. I. Struys M. M. Delfino A. Munoz H. (2008). The dynamic relationship between end-tidal sevoflurane concentrations, bispectral index, and cerebral state index in children. *Anesth. Analg.* 107 1573–1578. 10.1213/ane.0b013e318181ef88 18931214

[B21] Golińska A. K. (2013). Poincaré plots in analysis of selected biomedical signals. *Stud. Logic Grammar Rhetoric.* 35 117–127. 10.2478/slgr-2013-0031

[B22] Gross D. W. Gotman J. (1999). Correlation of high-frequency oscillations with the sleep-wake cycle and cognitive activity in humans. *Neuroscience* 94 1005–1018. 10.1016/s0306-4522(99)00343-7 10625043

[B23] Hayashi K. Mukai N. Sawa T. (2015a). Poincaré analysis of the electroencephalogram during sevoflurane anesthesia. *Clin. Neurophysiol.* 126 404–411. 10.1016/j.clinph.2014.04.019 24969375

[B24] Hayashi K. Yamada T. Sawa T. (2015b). Comparative study of Poincaré plot analysis using short electroencephalogram signals during anaesthesia with spectral edge frequency 95 and bispectral index. *Anaesthesia* 70 310–317. 10.1111/anae.12885 25271796

[B25] Jiang G. J. A. Fan S. Abbod M. F. Huang H. Lan J. Tsai F. (2015). Sample entropy analysis of EEG signals via artificial neural networks to model patients’ consciousness level based on anesthesiologists experience. *Biomed. Res. Int.* 2015:343478. 10.1155/2015/343478 25738152 PMC4337052

[B26] Kenwright D. A. Bernjak A. Draegni T. Dzeroski S. Entwistle M. Horvat M. (2015). The discriminatory value of cardiorespiratory interactions in distinguishing awake from anaesthetised states: a randomised observational study. *Anaesthesia* 70 1356–1368. 10.1111/anae.13208 26350998 PMC4989441

[B27] Khandoker A. H. Karmakar C. Brennan M. Palaniswami M. Voss A. (2013). “Quantitative Poincaré Plot,” in *Poincaré Plot Methods for Heart Rate Variability Analysis*, eds Khandoker A. H. Karmakar C. Brennan M. Palaniswami M. Voss A. (New York, NY: Springer Science+Business Media), 13–24. 10.1007/978-1-4614-7375-6

[B28] Kreuzer M. Stern M. A. Hight D. Berger S. Schneider G. Sleigh J. W. (2020). Spectral and entropic features are altered by age in the electroencephalogram in patients under sevoflurane anesthesia. *Anesthesiology* 132 1003–1016. 10.1097/ALN.0000000000003182 32108685 PMC7159998

[B29] Lee H. C. Ryu H. Yoon S. B. Yang S. M. Oh H. (2019). Data driven investigation of bispectral index algorithm. *Sci. Rep.* 9:13769. 10.1038/s41598-019-50391-x 31551487 PMC6760206

[B30] Leslie K. Chan M. T. Myles P. S. Forbes A. McCulloch T. J. (2010). Posttraumatic stress disorder in aware patients from the B-aware trial. *Anesth. Analg.* 110 823–828. 10.1213/ANE.0b013e3181b8b6ca 19861364

[B31] Lewis S. R. Pritchard M. W. Fawcett L. J. Punjasawadwong Y. (2019). Bispectral index for improving intraoperative awareness and early postoperative recovery in adults. *Cochrane Database Syst. Rev.* 9:CD003843 10.1002/14651858.CD003843 PMC6763215 31557307

[B32] Li D. Vlisides P. E. Kelz M. B. Avidan M. S. Mashour G. A. ReCCognition Study (2019). Dynamic cortical connectivity during general anesthesia in healthy volunteers. *Anesthesiology* 130 870–884. 10.1097/ALN.0000000000002656 30946055

[B33] Liang Z. Wang Y. Sun X. Li D. Voss L. J. Sleigh J. W. (2015). EEG entropy measures in anesthesia. *Front. Comput. Neurosci.* 9:16. 10.3389/fncom.2015.00016 25741277 PMC4332344

[B34] Luo C. Zou W. (2018). Cerebral monitoring of anaesthesia on reducing cognitive dysfunction and postoperative delirium: a systematic review. *J. Int. Med. Res.* 46 4100–4110. 10.1177/0300060518786406 30014748 PMC6166333

[B35] MacKenzie K. K. Britt-Spells A. M. Sands L. P. Leung J. M. (2018). Processed electroencephalogram monitoring and postoperative delirium: a systematic review and meta-analysis. *Anesthesiology* 129 417–427. 10.1097/ALN.0000000000002323 29912008 PMC6092196

[B36] Menon V. Freeman W. J. Cutillo B. A. Desmond J. E. Ward M. F. Bressler S. L. (1996). Spatio-temporal correlations in human gamma band electrocorticograms. *Electroencephalogr. Clin. Neurophysiol.* 98 89–102. 10.1016/0013-4694(95)00206-5 8598178

[B37] Messner M. Beese U. Romstock J. Dinkel M. Tschaikowsky K. (2003). The bispectral index declines during neuromuscular block in fully awake persons. *Anesth. Analg.* 97 488–491. 10.1213/01.ane.0000072741.78244.c0 12873942

[B38] Morimoto Y. Hagihira S. Koizumi Y. Ishida K. Matsumoto M. Sakabe T. (2004). The relationship between bispectral index and electroencephalographic parameters during isoflurane anesthesia. *Anesth. Analg.* 98 1336–1340. 10.1213/01.ane.0000105867.17108.b6 15105211

[B39] Musizza B. Stefanovska A. McClintock P. V. E. Palus M. Petrovcic J. Ribaric S. (2007). Interactions between cardiac, respiratory and EEG-delta oscillations in rats during anaesthesia. *J. Physiol.* 580 315–326. 10.1113/jphysiol.2006.126748 17234691 PMC2075429

[B40] Myles P. S. Leslie K. McNeil J. Forbes A. Chan M. T. (2004). Bispectral index monitoring to prevent awareness during anaesthesia: the B-Aware randomised controlled trial. *Lancet* 363 1757–1763. 10.1016/S0140-6736(04)16300-9 15172773

[B41] Ni K. Cooter M. Gupta D. K. Thomas J. Hopkins T. J. Miller T. E. (2019). Paradox of age: older patients receive higher age-adjusted minimum alveolar concentration fractions of volatile anaesthetics yet display higher bispectral index values. *Brit. J. Anaesth.* 123 288–297. 10.1016/j.bja.2019.05.040 31279479 PMC7104362

[B42] Purdon P. Pavone K. J. Akeju O. Smith A. C. Sampson A. L. Lee J. (2015). The ageing brain: age-dependent changes in the electroencephalogram during propofol and sevoflurane general anaesthesia. *Brit. J. Anaesth.* 115 i46–i57. 10.1093/bja/aev213 26174300 PMC4501918

[B43] Purdon P. L. Sampson A. Pavone K. J. Brown E. N. (2015). Clinical electroencephalography for anesthesiologists. Part I: background and basic signatures. *Anesthesiology* 123 937–960. 10.1097/ALN.0000000000000841 26275092 PMC4573341

[B44] Radtke F. M. Franck M. Lendner J. Krüger S. Wernecke K. D. Spies C. D. (2013). Monitoring depth of anaesthesia in a randomized trial decreases the rate of postoperative delirium but not postoperative cognitive dysfunction. *Br. J. Anaesth.* 110 i98–i105. 10.1093/bja/aet055 23539235

[B45] Rampil I. J. (1998). A primer for EEG signal processing in anesthesia. *Anesthesiology* 89 980–1002. 10.1097/00000542-199810000-00023 9778016

[B46] Rigouzzo A. Khoy-Ear L. Laude D. Louvet N. Moutard M. L. (2019). EEG profiles during general anesthesia in children: A comparative study between sevoflurane and propofol. *Paediatr. Anaesth.* 29 250–257. 10.1111/pan.13579 30614153

[B47] Sigl J. C. Chamoun N. G. (1994). An introduction to bispectral analysis for the electroencephalogram. *J. Clin. Monit.* 10 392–404. 10.1007/BF01618421 7836975

[B48] Schuller P. Newell S. Strickland P. Barry J. (2015). Response of bispectral index to neuromuscular block in awake volunteers. *Brit. J. Anaesth.* 115 i95–i103. 10.1093/bja/aev072 26174308

[B49] Sciusco A. Standing J. F. Sheng Y. Raimondo P. Cinnella G. Dambrosio M. (2017). Effect of age on the performance of bispectral and entropy indices during sevoflurane pediatric anesthesia: a pharmacometric study. *Paediatr. Anaesth.* 27 399–408. 10.1111/pan.13086 28211134

[B50] Sleigh J. W. Steyn-Ross D. A. Steyn-Ross M. L. Williams M. L. Smith P. (2001). Comparison of changes in electroencephalographic measures during induction of general anaesthesia: influence of the gamma frequency band and electromyogram signal. *Br. J. Anaesth.* 86 50–58. 10.1093/bja/86.1.50 11575410

[B51] Su C. Liang Z. Li X. Li D. Li Y. Ursino M. (2016). A comparison of multiscale permutation entropy measures in on-line depth of anesthesia monitoring. *PLoS One* 11:e0164104. 10.1371/journal.pone.0164104 27723803 PMC5056744

[B52] Tang C. J. Jin Z. Sands L. P. Pleasants D. Tabatabai S. Hong Y. (2020). ADAPT-2: a randomized clinical trial to reduce intraoperative EEG suppression in older surgical patients undergoing major noncardiac surgery. *Anesth. Analg.* 131 1228–1236. 10.1213/ANE.0000000000004713 32925344 PMC7599075

[B53] Tulppo M. P. Makikallio T. H. Takala T. E. Seppanen T. Huikuri H. V. (1996). Quantitative beat-to-beat analysis of heart rate dynamics during exercise. *Am. J. Physiol.* 271 H244–H252. 10.1152/ajpheart.1996.271.1.H244 8760181

[B54] Wang F. Zhang J. Yu J. Tian M. Cui X. Wu A. (2019). Variation of bispectral index in children aged 1-12 years under propofol anesthesia: an observational study. *BMC Anesthesiol.* 19:145. 10.1186/s12871-019-0815-6 31390975 PMC6686421

[B55] Whitlock E. L. Avidan M. S. (2020). Three blind mice: a tail of discordant trials. *Br. J. Anaesth.* 124 121–125. 10.1016/j.bja.2019.09.035 31676036

[B56] Wildes T. S. Mickle A. M. Abdallah A. B. Maybrier H. R. Oberhaus J. Budelier T. P. (2019). Effect of electroencephalography-guided anesthetic administration on postoperative delirium among older adults undergoing major surgery: The ENGAGES randomized clinical trial. *JAMA* 321 473–483. 10.1001/jama.2018.22005 30721296 PMC6439616

