# Supplementary figures and images for: Poincaré Plot Area of Gamma-Band EEG as a Measure of Emergence From Inhalational General Anesthesia
Source: Front Physiol. 2021 Feb 9;12:627088. doi: 10.3389/fphys.2021.627088 (PMC7900422; doi:10.3389/fphys.2021.627088)

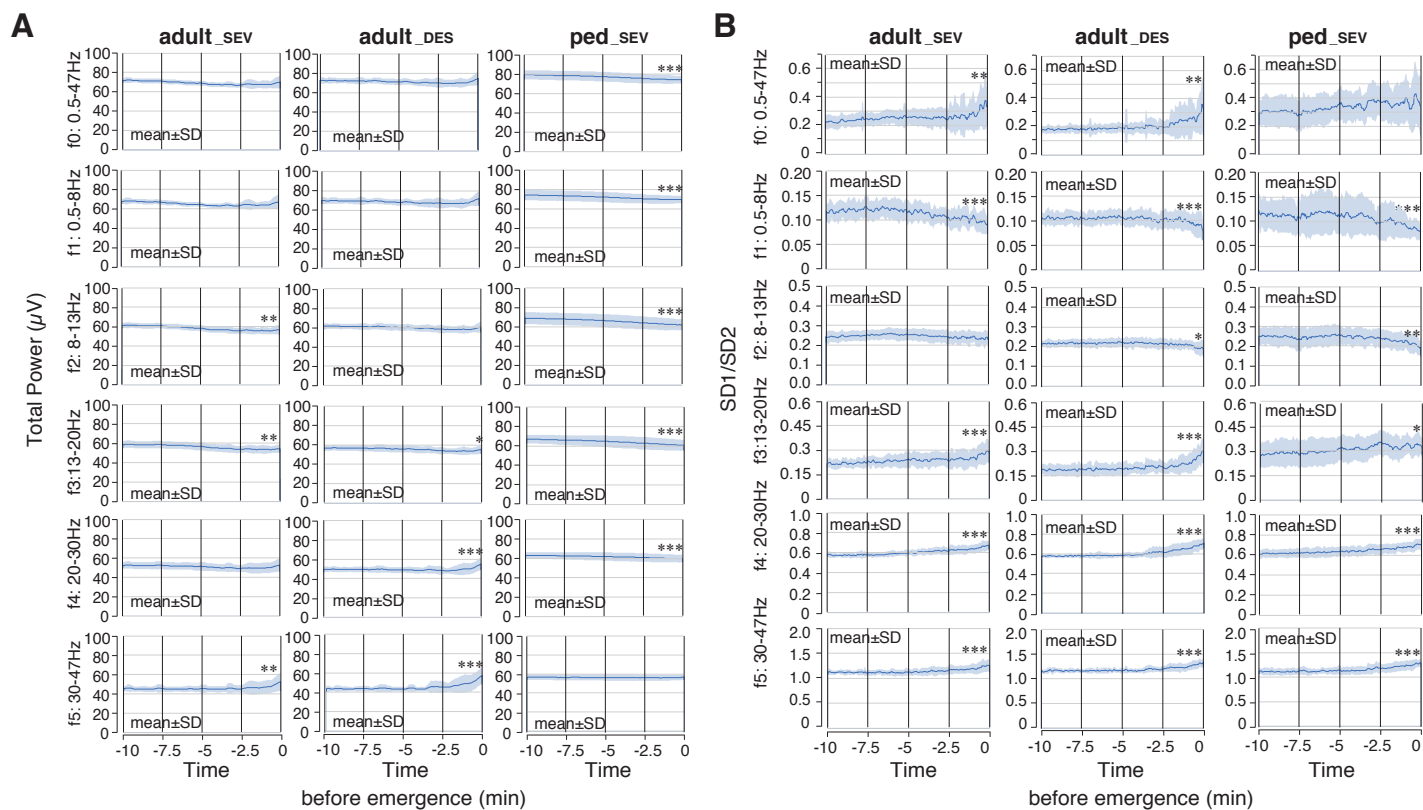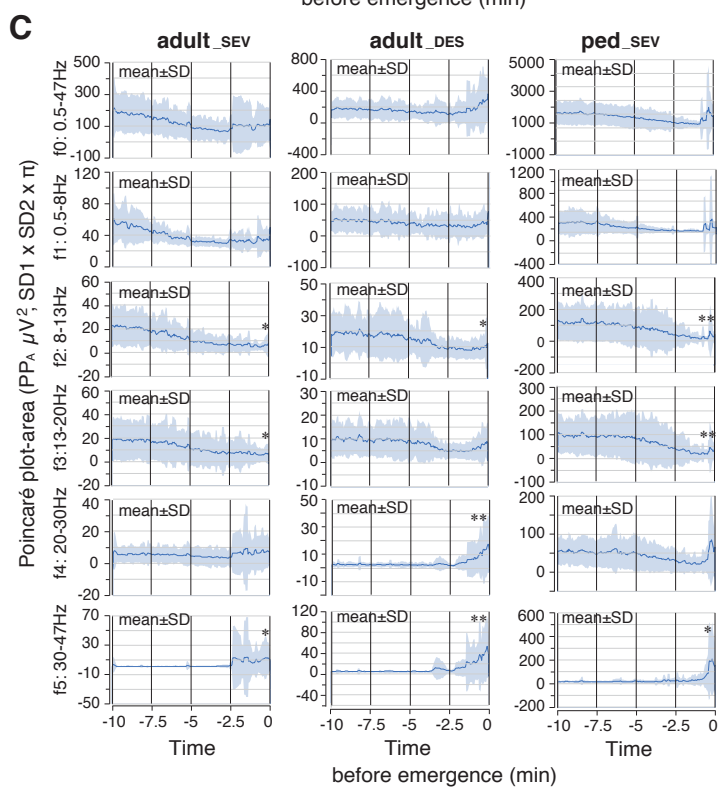

Supplement: Supplementary Figure 1 — Total EEG power and Poincaré plot parameters throughout the time course from the hypnotic condition to the awake state in anesthetized patients. (A) Total preprocessed EEG power after passing through one of five bandpass filters, (B) SD1/SD2 of the Poincaré plot of the preprocessed EEG signal after passing through one of six bandpass filters, (C) Area of the Poincaré plot (PPA) of the processed EEG signal after passing through one of six bandpass filters. [file Image_1.PDF]

# A

## 1)

**adult\_sev**  
(n=20)

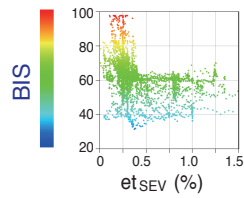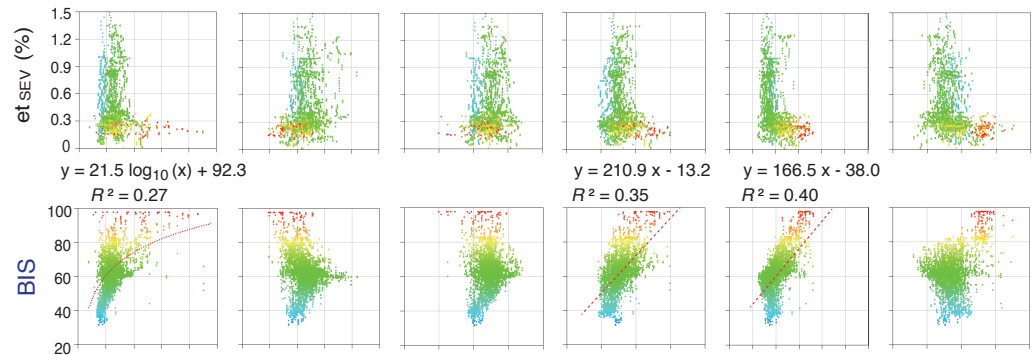

## 2)

**adult\_DES**  
(n=20)

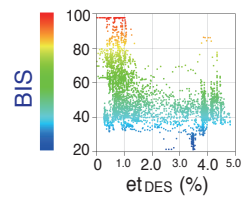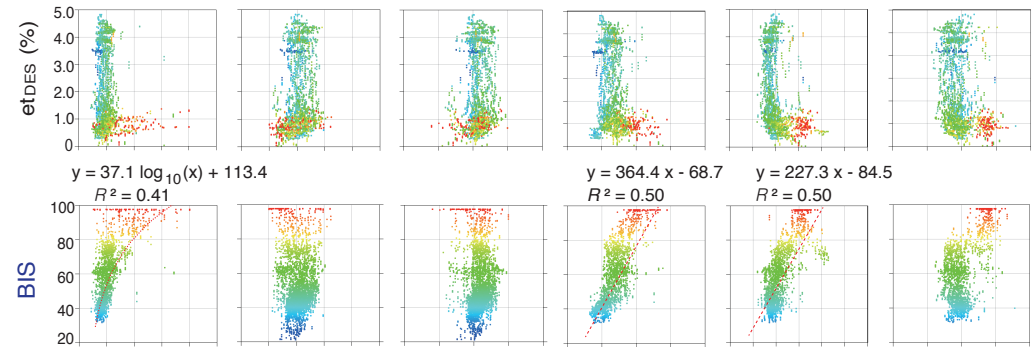

## 3)

**ped\_sev**  
(n=20)

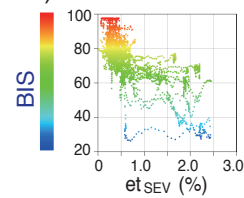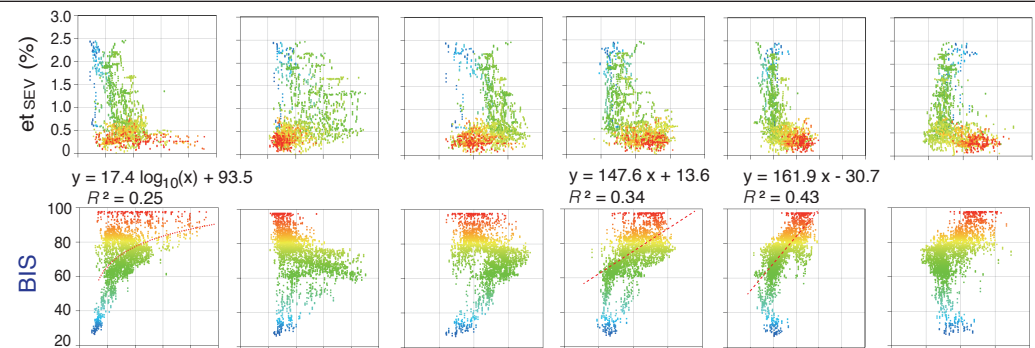

## 4)

**all\_combined**  
(n=60)

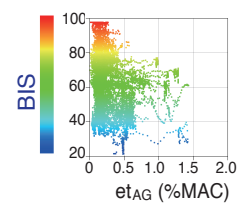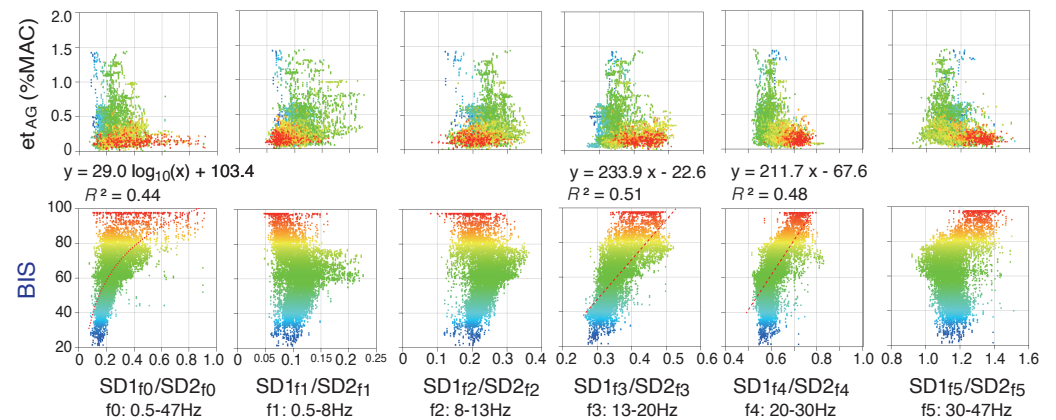

# B

## 1)

**adult\_sev**  
(n=20)

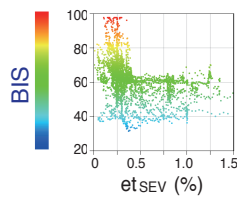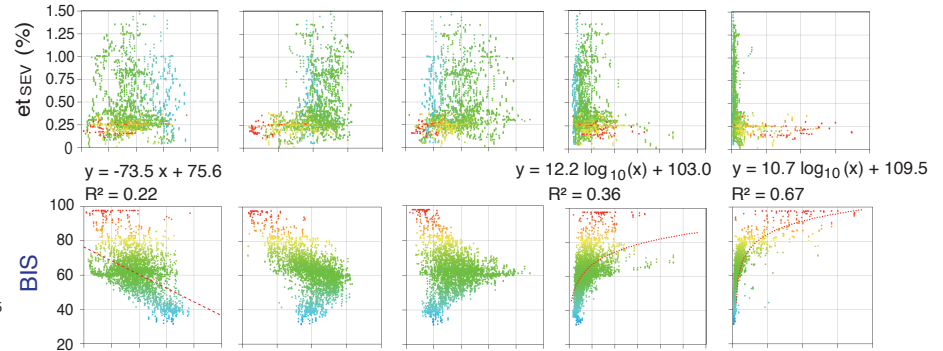

## 2)

**adult\_DES**  
(n=20)

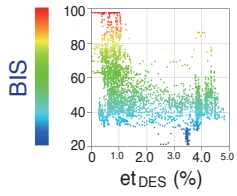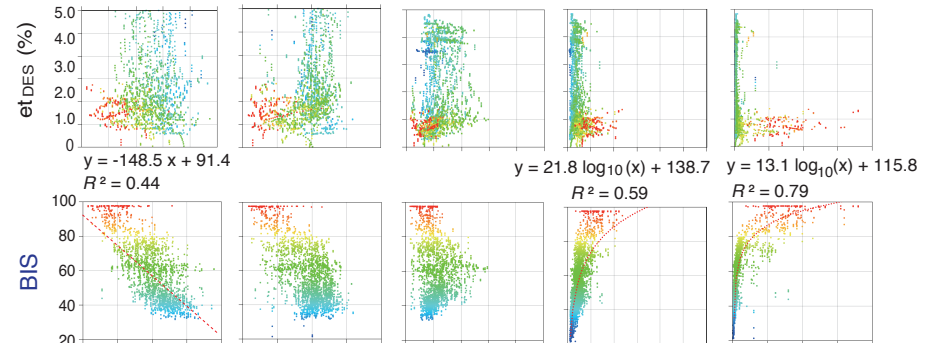

## 3)

**ped\_sev**  
(n=20)

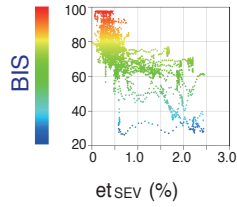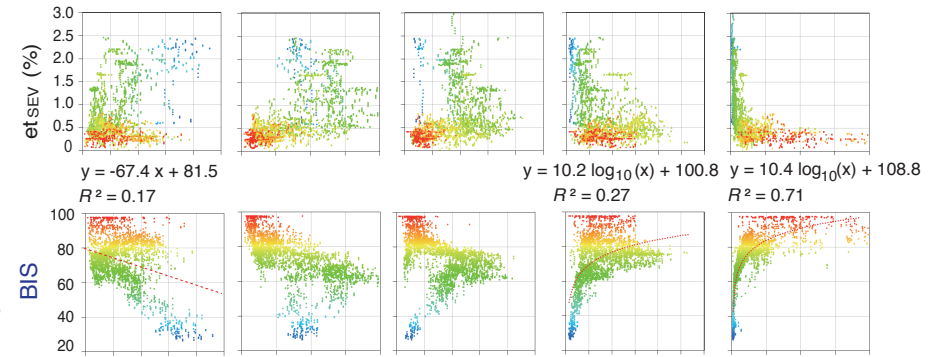

## 4)

**all\_combined**  
(n=60)

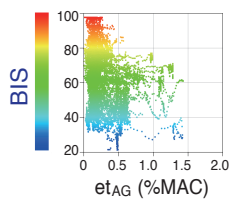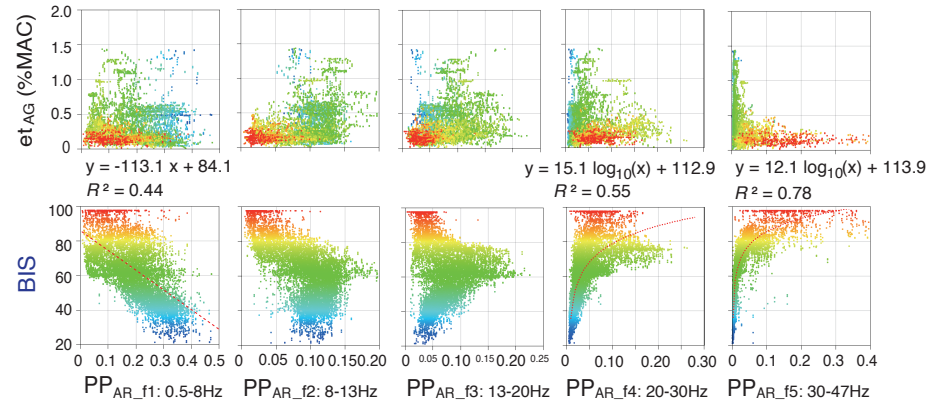

Supplement: Supplementary Figure 2 — Relationships of Poincaré plot parameters with BIS and etAG. (A) The relationships of Poincaré plot SD1/SD2 with BIS and etAG. (B) The relationships of Poincaré plot Poincaré plot area ratio (PPAR) with BIS and etAG. [file Image_2.PDF]

**A** **adult\_SEV**

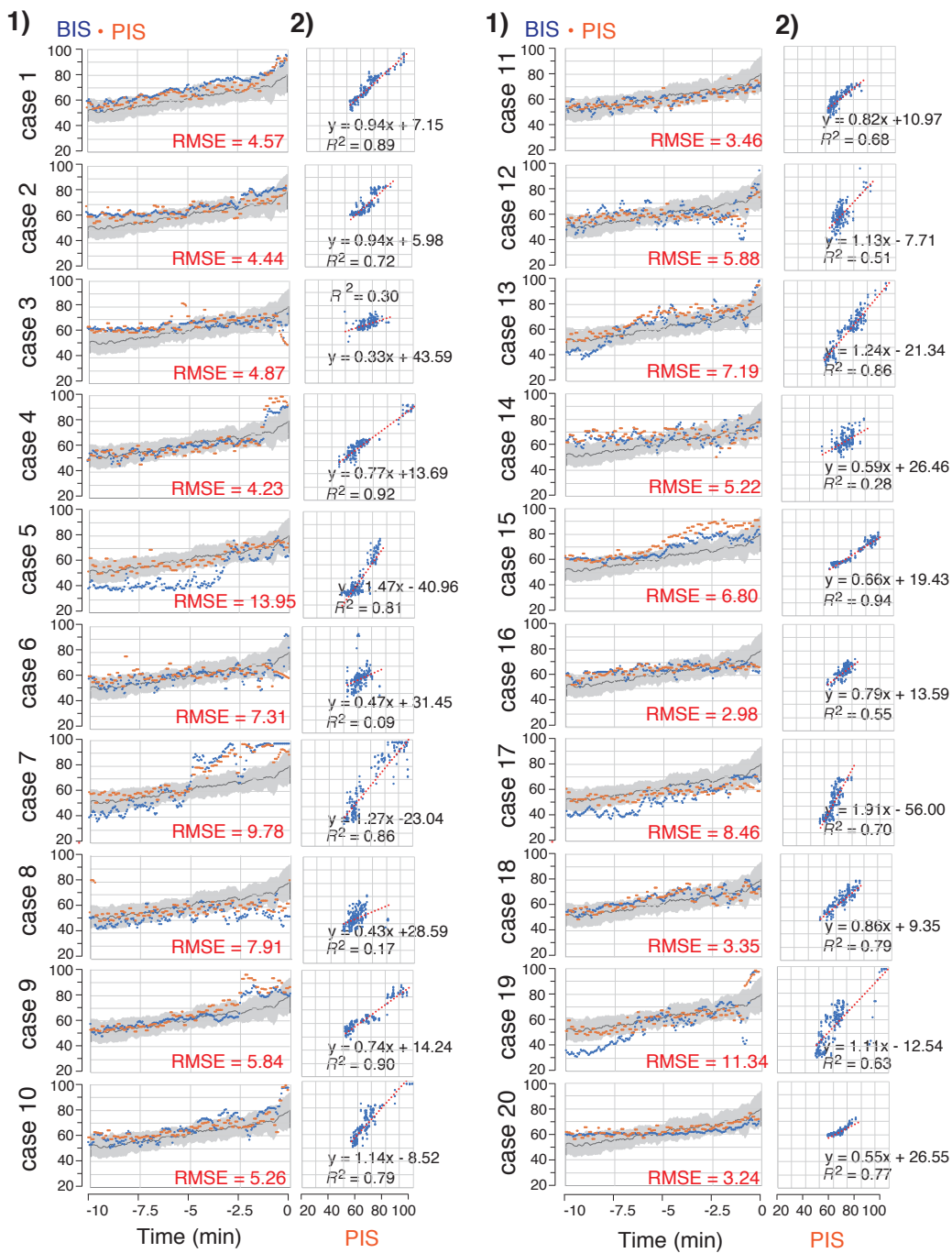

### 3) averages of 20 cases

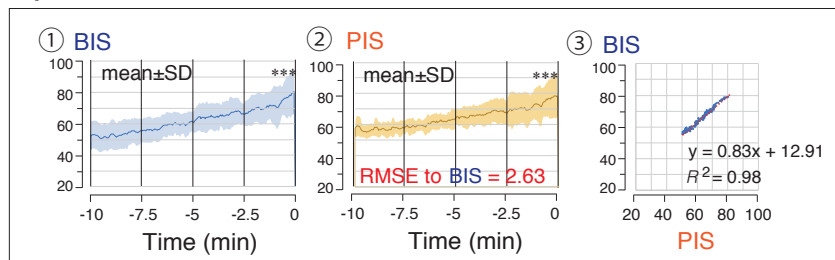

## B adult\_DES

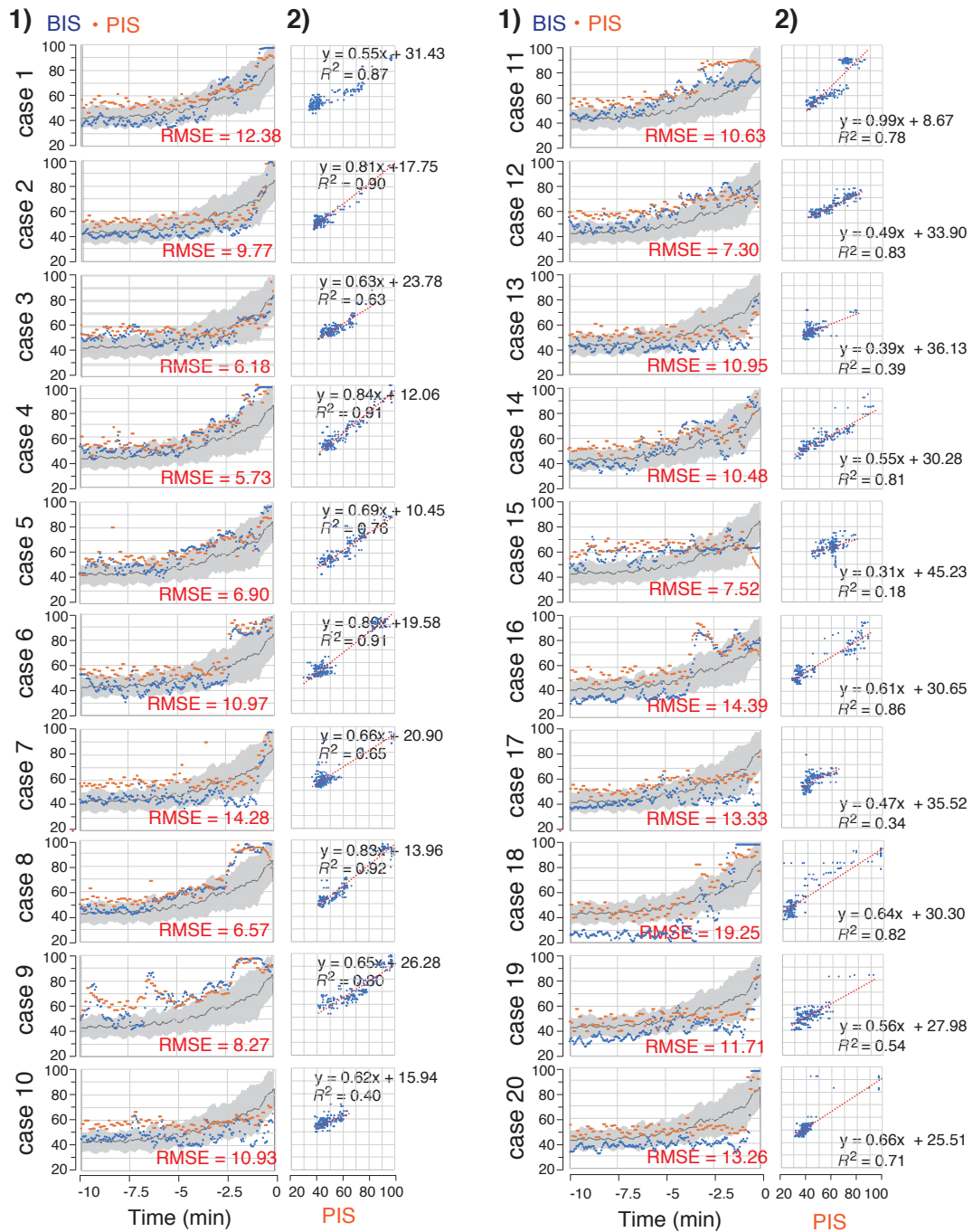

### 3) averages of 20 cases

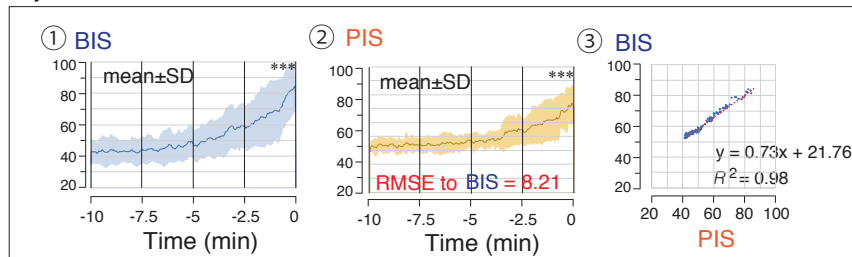

## C ped\_sev

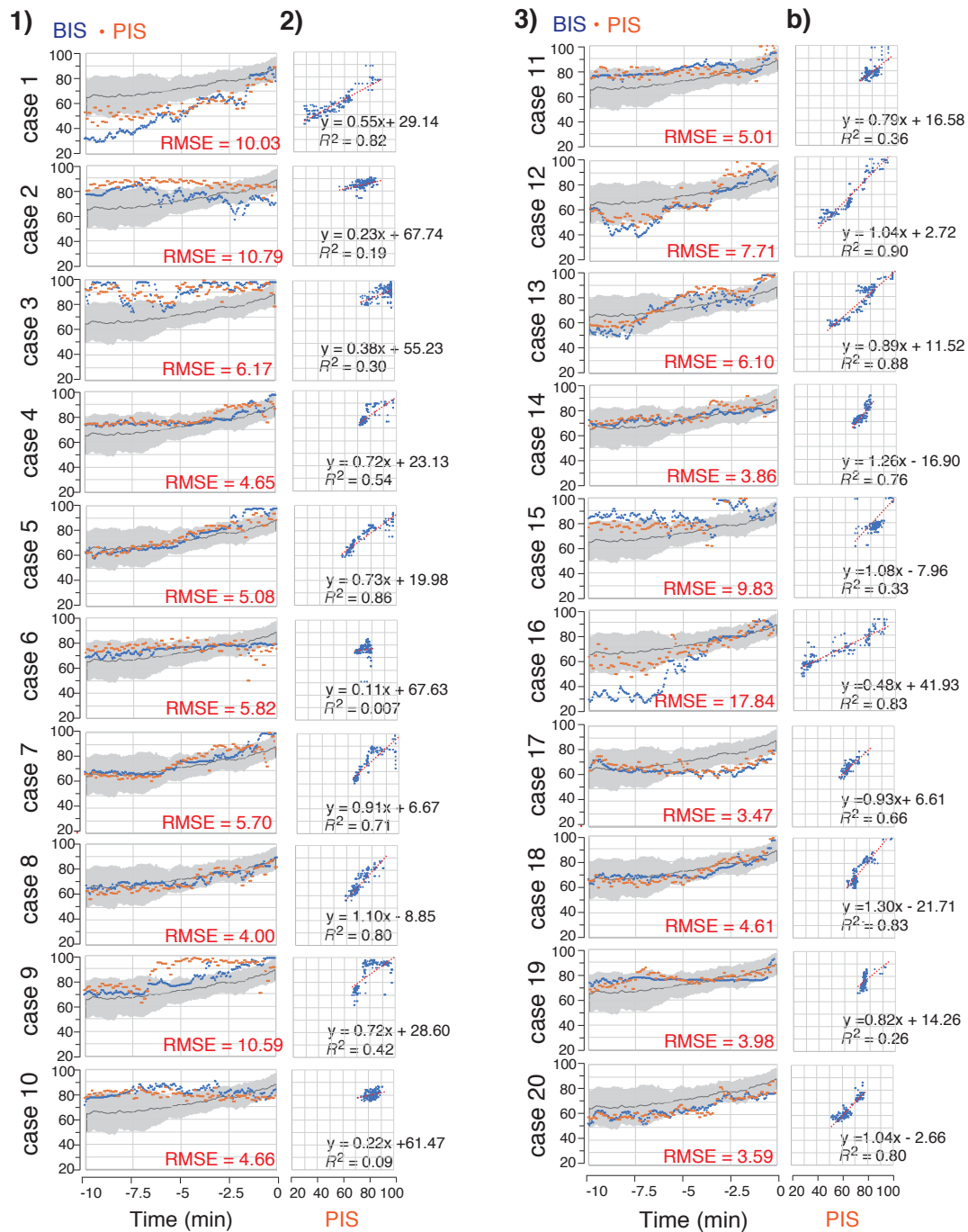

### 3) averages of 20 cases

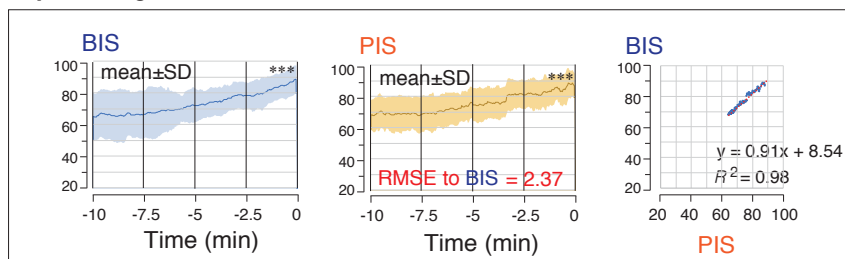

Supplement: Supplementary Figure 3 — Case-by-case plots of time-course change of PIS and BIS, and linear regressions between PIS and BIS. (A) Adult sevoflurane (adult_SEV) group. (B) Adult sevoflurane (adult_DES) group. (C) Adult sevoflurane (ped_SEV) group. [file Image_3.pdf]
