# Supplementary material for: Poincaré Plot Area of Gamma-Band EEG as a Measure of Emergence From Inhalational General Anesthesia
Source: Front Physiol. 2021 Feb 9;12:627088. doi: 10.3389/fphys.2021.627088 (PMC7900422; doi:10.3389/fphys.2021.627088)
Supplement: Supplementary Table 1 — Detailed information about patients and anesthesia management. [file Table_1.PDF]

SUPPLEMENTARY TABLE S1 | Detailed information about patients and anesthesia management

Group	No.	Age	Gender	Ht (cm)	BW (kg)	Surgery	Surgery time (min)	Anesthesia time (min)	Total dosage			The end of the surgery (min:sec before extubation)
									Fentanyl (μg/kg)	Rocuronium (mg/kg)	Sugammadex (mg/kg) & (timing, min before extubation)	
Adult sevoflurane (adult$_{SEV}$)	1	54	F	157	61	arthrodesis of the left hallux metatarsophalangeak joint	173	241	8.2	1.3	3.3 (−10)	−30
	2	31	F	160	45	open reduction and internal fixation to repair left foot joint fracture	108	174	7.8	1.4	4.5 (−7)	−24
	3	66	F	147	56	modified Mann procedure	91	142	4.5	1.3	3.6 (−8)	−22
	4	29	F	165	80	removal of screws	28	80	2.5	0.8	2.5 (−9)	−22
	5	79	M	167	59	transurethral resection of bladder tumor	77	132	3.4	1.2	3.4 (−8)	−15
	6	63	M	177	72	ureteroscopy	40	87	2.8	0.7	2.1 (−6)	−18
	7	66	M	157	59	right lower tibia osteotomy	164	227	6.4	1.5	3.4 (−8)	−21
	8	54	M	183	84	anterior transposition of left ulnar nerve	99	168	3.0	1.1	2.4 (−8)	−22
	9	60	F	156	62	medial displacement left calcaneal osteotomy	129	188	3.2	1.5	3.2 (−11)	−20
	10	56	F	158	50	modified Mann procedure	71	125	3.0	0.8	2.0 (−7)	−21
	11	65	F	161	69	cheilectomy	57	108	2.2	0.6	2.2 (−6)	−18
	12	78	F	148	52	left thumb arthroplasty	134	203	3.9	1.2	2.9 (−13)	−24
	13	29	F	160	57	laparoscopic ovarian tumor resection	66	103	4.9	1.1	3.5 (−9)	−13
	14	69	F	159	65	right mastectomy and sentinel lymph node biopsy	107	160	3.8	1.2	3.1 (−6)	−18
	15	61	F	158	44	right total hip arthroplasty	94	174	6.8	1.1	4.5 (−7)	−23
	16	59	M	172	77	transurethral resection of bladder tumor	76	122	2.0	1.1	2.6 (−13)	−20
	17	75	M	165	67	resection of left thoracic soft tissue tumor	52	122	3.0	0.8	3.3 (−10)	−15
	18	43	M	167	62	resection of left femoral soft tissue tumor	169	254	4.0	2.7	3.2 (−9)	−18
	19	69	F	148	47	right total hip arthroplasty	86	172	5.4	0.6	2.2 (−11)	−28
	20	24	M	175	62	resection of right patellar soft tissue tumor	131	188	4.1	0.5	2.1 (−9)	−19
	range	24 – 79	M/F = 8/12	147 - 183	44 - 84		28 - 173	80 - 254	2.0 - 8.2	0.5 - 2.7	2.0 - 4.5 (−13 to −6)	−30 to −17
	mean±SD	56.5 ± 16.8		161.9 ± 9.7	61.5 ± 11.2		97.6 ± 42.0	158.5 ± 49.6	4.23 ± 1.81	1.12 ± 0.49	2.98 ± 0.73 (−8.8 ± 2.1)	−20.6 ± 4.2
	95%CI	48.6 - 64.4	157.5 - 166.5		56.3 - 66.7		78.0 - 117.3	135.3 - 181.7	3.4 - 5.1	0.9 - 1.3	2.7 - 3.3 (-9.7 - -7.8)	-22.5 - 18.6
Adult desflurane (adult$_{DES}$)	1	65	F	158	61	partial thyroidectomy	150	230	4.9	1.5	3.3 (−6)	−13
	2	71	M	172	65	transurethral resection of bladder tumor	121	160	2.3	1.6	3.1 (−3)	−12
	3	52	F	151	56	left total hip arthroplasty	137	235	4.5	1.4	3.6 (−4)	−23
	4	38	F	167	62	laparoscopic ovarian tumor resection	99	137	4.0	1.1	3.2 (−4)	−11
	5	28	F	165	55	cervical conization	41	82	1.8	0.7	1.8 (−3)	−10
	6	41	F	161	57	hysteroscopic surgery	28	77	1.8	1.1	3.5 (−5)	−10
	7	59	M	163	64	transurethral ureterolithotomy	195	233	3.9	2.0	3.1 (−3)	−12
	8	48	M	163	51	left total hip arthroplasty	100	187	4.9	1.3	3.0 (−5)	−18
	9	50	F	157	79	left total hip arthroplasty	93	191	4.4	1.3	2.5 (−4)	−26
	10	65	F	157	57	right mastectomy and lymph node dissection	167	218	4.4	1.8	3.5 (−5)	−12
	11	73	M	173	65	laparoscopic cholecystectomy	74	152	5.4	1.0	1.9 (−9)	−23
	12	56	F	160	66	resection of right forearm soft tissue tumor	24	72	2.3	0.8	3.0 (−7)	−14
	13	72	F	154	47	resection of right upper arm malignant soft tissue tumor	214	302	6.4	1.5	4.2 (−4)	−23
	14	43	F	182	62	left mastectomy and sentinel lymph node biopsy	119	167	4.1	1.3	3.3 (−8)	−15
	15	33	M	171	78	resection of left forearm soft tissue tumor	47	108	2.6	0.8	2.6 (−12)	−21
	16	77	F	153	55	right mastectomy and sentinel lymph node biopsy	172	219	1.8	1.7	3.7 (−6)	−15
	17	59	M	171	64	resection of right foot tumor margin	127	193	3.1	1.1	3.1 (−8)	−15
	18	60	F	151	46	left mastectomy and lymph node dissection	135	190	2.2	1.7	4.4 (−3)	−18
	19	52	F	150	68	artificial joint replacement of the left finger	173	246	3.7	1.3	2.9 (−8)	−23
	20	59	M	171	95	right thumb amputation	66	110	3.2	1.1	2.1 (−11)	−13
	range	28 - 77	M/F = 7/13	150 - 182	46 - 95		24 - 214	72 - 302	1.8 - 6.4	0.7 - 2.0	1.8 - 4.4 (−12 to −3)	−26 to −10
	mean±SD	55.1 ± 13.7		162.5 ± 8.9	62.6 ± 11.5		114.1 ± 55.6	175.5 ± 63.1	3.58 ± 1.33	1.28 ± 0.35	3.09 ± 0.68 (−5.9 ± 2.7)	−16.4 ± 5.1
	95%CI	48.6 - 61.5	158.4 - 166.7		57.3 - 68.0		88.1 - 140.1	145.9 - 205.0	3.0 - 4.2	1.1 - 1.5	2.8 - 3.4 (-7.2 - -4.6)	-18.7 - 14.0
Pediatric sevoflurane (ped$_{SEV}$)	1	4	M	107	17	laparoscopic inguinal hernia radical surgery	43	71	1.7	1.2	3.5 (−1)	−5
	2	1	F	87	10	surplus scissor cutting	103	163	2.9	1.5	2.9 (−11)	−16
	3	1	M	77	11	umbilical hernia radical surgery	89	199	3.5	1.5	1.8 (−13)	−13
	4	2	F	85	12	testicular fusion	56	99	2.1	1.3	3.3 (−2)	−5
	5	5	M	106	18	scrotal edema radical surgery	28	83	0.0	1.1	2.2 (−3)	−9
	6	6	M	120	24	scrotal edema radical surgery	120	179	6.3	1.7	8.3 (−12)	−18
	7	2	M	85	11	posthioplasty	42	76	2.8	1.4	3.8(−3)	−7
	8	3	M	91	12	broviac catheter insertion	40	87	2.4	1.6	4.9 (−3)	−10
	9	6	F	115	21	umbilical hernia radical surgery	72	113	2.0	1.2	3.9 (−7)	−12
	10	8	F	131	24	median cervical cyst radical surgery	61	100	2.9	0.8	2.1 (−8)	−12
	11	7	F	127	27	laparoscopic inguinal hernia radical surgery	167	402	3.7	1.9	1.5 (−1)	−37
	12	8	M	129	28	laparoscopic inguinal hernia radical surgery	113	152	1.8	1.6	1.5 (−4)	−12
	13	3	M	98	17	scrotal edema radical surgery	64	104	2.9	1.8	4.1 (−8)	−13
	14	8	M	133	29	scrotal edema radical surgery	55	98	1.8	1.2	4.2 (−11)	−15
	15	10	M	143	32	subcutaneous tunnel type central venous catheterization	95	160	4.8	1.9	3.8 (−16)	−24
	16	2	M	75	8	subcutaneous tunnel type central venous catheterization	45	90	1.2	1.2	3.7 (−6)	−15
	17	6	M	110	20	left orchidopexy	67	114	2.0	1.5	4.1 (−11)	−15
	18	4	M	99	16	bilateral orchidopexy	121	165	2.5	1.9	3.8 (−8)	−11
	19	2	F	87	11	laparoscopic inguinal hernioplasty	75	127	1.8	1.8	0.0 (0)	−15
	20	3	M	98	14	right scrotal hydrocele radical surgery	36	72	1.5	1.1	0.0 (0)	−8
	range	1月10日	M/F = 14/6	75 - 143	8 - 32		28 - 167	71 - 402	0 - 6.3	0.8 - 1.9	0.0 - 8.3 (−16 to −1)	−37 to −5
	mean±SD	4.6 ± 2.7		105.2 ± 20.2	18.1 ± 7.1		74.6 ± 35.9	132.7 ± 74.1	2.53 ± 1.34	1.46 ± 0.31	3.17 ± 1.83 (−6.4 ± 4.8)	−13.6 ± 7.1
	95%CI	3.3 - 5.8	95.7 - 114.6		14.7 - 21.5		57.8 - 91.4	98.0 - 167.4	1.9 - 3.2	1.3 - 1.6	2.3 - 4.0 (8.6 - -4.2)	-16.9 - -10.3
range		1 - 79	M/F = 29/31	75 - 183	8 - 95		24 - 214	71 - 402	0 - 8.2	0.5 - 2.7	0.0 - 8.3 (−16 to −1)	−37 to −5
mean±SD		38.7 ± 27.3		143.2 ± 30.4	47.4 ± 23.2		95.4 ± 47.4	155.6 ± 64.5	3.44 ± 1.64	1.29 ± 0.41	3.08 ± 1.19 (−7.0 ± 3.6)	−16.8 ± 6.2
95% Confidence Interval		31.6 - 45.8	135.4 - 151.1		41.4 - 53.4		83.2 - 107.7	138.9 - 172.2	3.0 - 3.9	1.2 - 1.4	2.8 - 3.4 (-7.9 - -6.1)	-18.4 - -15.2

95%CI: 95% confidence Interval

M: male, F: female
