# Supplementary material for: Poincaré Plot Area of Gamma-Band EEG as a Measure of Emergence From Inhalational General Anesthesia
Source: Front Physiol. 2021 Feb 9;12:627088. doi: 10.3389/fphys.2021.627088 (PMC7900422; doi:10.3389/fphys.2021.627088)
Supplement: Supplementary Table 2 — Comparison of changes in EEG parameters. (A) The comparison between three groups. (B) The comparison in the all_combined group. [file Table_2.PDF]

Supplementary Table 2S

SUPPLEMENTARY TABLE S2_A. Comparison of changes in EEG parameters. The comparison between the three groups.

item/group	adult$_{_SEV}$ (n=20)			adult$_{_DES}$ (n=20)			ped$_{_SEV}$ (n=20)		
	10 min before emergence (EM_{-10})	at time of emergence (EM_0)	*p value*	10 min before emergence (EM_{-10})	at time of emergence (EM_0)	*p value*	10 min before emergence (EM_{-10})	at time of emergence (EM_0)	*p value*
et_{AG} (%)	0.97±0.32	0.17±0.10	<0.0001	3.71±0.84	0.68±0.42	<0.0001	1.34±0.75	0.27±0.16	<0.0001
BIS	52.9±8.9	80.5±13.8	<0.0001	41.9±8.8	84.7±16.4	<0.0001	65.4±14.9	88.4±9.1	<0.0001
SEF_{95} (Hz)	16.8±2.5	21.7±4.4	0.0001	13.6±1.9	20.3±4.6	<0.0001	20.8±4.5	24.1±3.3	0.0126
RelativeBetaRatio	−1.53±0.19	−1.41±0.24	0.0404	−1.60±0.20	−1.30±0.28	0.001**	−1.49±0.32	−1.30± 0.24	0.0071
EMGlow (dB)	27.2±1.76	45.06±11.76	<0.0001	27.47±1.59	54.11±5.58	<0.0001	33.67±5.8	52.75± 12.00	<0.0001
Total power (μV)									
f0: 0.5–47Hz	71.5±2.4	70.0±6.8	0.3739	73.3±3.7	74.6±6.5	0.2405	79.3±4.8	74.3±3.8	<0.0001
f1: 0.5–8Hz	67.7±2.7	66.9±7.6	0.6754	70.6±4.0	72.3±6.9	0.1596	74.4±5.6	69.6±3.7	0.0008
f2: 8–13Hz	61.1±2.8	56.7±5.1	0.0063	61.9±3.4	60.3±5.3	0.0637	68.9±6.2	62.1±5.6	<0.0001
f3: 13–20Hz	58.5±3.8	54.3±4.6	0.0035	57.3±3.3	55.5±4.6	0.0362	67.1±4.4	60.8±5.6	<0.0001
f4: 20–30Hz	52.2±3.6	52.6±6.8	0.7694	50.0± 3.2	55.2±6.2	0.0003	63.1±3.4	59.7±3.9	0.0009
f5: 30–47Hz	45.1±2.8	52.3±9.4	0.0036	44.1±3.7	57.5±8.3	<0.0001	56.7±2.9	56.6±2.7	0.8187
SD1/SD2									
f0: 0.5–47Hz	0.23±0.05	0.34±0.14	0.0035	0.18±0.03	0.31±0.19	0.0047	0.30±0.09	0.34±0.22	0.4989
f1: 0.5–8Hz	0.12±0.02	0.09±0.02	0.0003	0.11±0.01	0.08±0.02	<0.0001	0.12±0.04	0.08±0.02	0.0003
f2: 8–13Hz	0.24±0.03	0.23±0.04	0.0003	0.22±0.02	0.19±0.04	0.0185	0.25±0.05	0.19±0.05	0.0042
f3: 13–20Hz	0.34±0.02	0.39±0.05	0.0003	0.32±0.02	0.40±0.05	<0.0001	0.38±0.04	0.41±0.04	0.0423
f4: 20–30Hz	0.58±0.02	0.67±0.04	<0.0001	0.57±0.02	0.71±0.05	<0.0001	0.61±0.04	0.70±0.05	<0.0001
f5: 30–47Hz	1.12±0.05	1.26±0.13	0.0001	1.16±0.04	1.33±0.08	<0.0001	1.12±0.06	1.28±0.08	<0.0001
PP_A (μV^2)									
f0: 0.5–47Hz	181.5±143.4	141.2±189.6	0.4867	176.1±106.6	350.5±548.7	0.1321	1117.9±799.3	898.3±894.4	0.4392
f1: 0.5–8Hz	34.8±30.1	29.7±71.9	0.4867	50.7±33.2	77.6±174.5	0.1321	185.1±215.9	96.8±133.4	0.1736
f2: 8–13Hz	21.9±18.9	8.0±11.7	0.0186	19.8±13.6	12.4±16.2	0.0316	127.0±114.5	40.1±66.5	0.0088
f3: 13–20Hz	17.5±16.9	7.0±8.7	0.0157	10.3±7.3	8.4±7.8	0.3504	105.1±79.3	44.7±61.0	0.0123
f4: 20–30 Hz	5.7±5.7	7.1±8.1	0.4030	2.6±1.6	13.0±14.9	0.0054	56.3±34.1	66.6±89.0	0.6443
f5: 30–47 Hz	1.1±1.0	10.2±14.0	0.0115	0.7±0.3	31.8±36.7	0.0012	15.3±.7.5	155.7±269.0	0.031
PP_{AR}									
f1: 0.5–8Hz	0.210±0.076	0.156±0.093	0.0115	0.285±0.059	0.202±0.113	0.0018	0.142±0.093	0.134±0.077	0.7842
f2: 8–13Hz	0.115±0.016	0.067±0.036	<0.0001	0.110±0.017	0.051±0.035	<0.0001	0.094±0.041	0.044±0.026	0.0001
f3: 13–20Hz	0.089±0.031	0.063±0.033	0.0025	0.059±0.020	0.035±0.016	0.0004	0.097±0.045	0.047±0.030	<0.0001
f4: 20–30Hz	0.030±0.013	0.061±0.042	0.0037	0.016±0.007	0.044±0.027	0.0003	0.070±0.046	0.067±0.036	0.8037
f5: 30–47Hz	0.007±0.003	0.078±0.074	0.0005	0.004±0.002	0.098±0.096	0.0003	0.025±0.031	0.124±0.113	0.0018**
PIS	57.4±5.1	78.6±13.3	<0.0001	52.5±4.2	81.4±13.7	<0.0001	68.2±9.0	86.7±7.9	<0.0001

Data of EM_{-10} and EM_0 are shown as mean±SD. Student's paired *t*-test was used for statistical comparisons between the data from two time points, EM_{-10} and EM_0. $^{*}p<0.05$, $^{**}p<0.01$, and $^{***}p<0.001$. adult$_{_SEV}$, adult patients anesthetized with sevoflurane; adult$_{_DES}$, adult patients anesthetized with desflurane; ped$_{_SEV}$, pediatric patients anesthetized with sevoflurane. BIS, bispectral index; EEG, electroencephalography; EM_{-10}, 10 min before emergence; EM_0, at the time of emergence; et_{AG}, end-tidal anesthetic gas concentration; EMGlow, a band power for the range 70-110 Hz value in decibel (dB) with respect to 0.0001 μV^2; PIS, Poincaré plot-area integrated score; PP_A, Poincaré plot area; PP_{AR}, Poincaré plot-area ratio; SEF_{95}, spectral edge frequency below which 95% of the power of a given signal is located.

SUPPLEMENTARY TABLE S2_B. Comparison of changes in EEG parameters. The comparison in the all$_{_combined}$ group.

item/group	all$_{_combined}$ (n=60)		
	10 min before emergence (EM_{-10})	at time of emergence (EM_0)	*p value*
et_{AG} (%MAC)	0.61±0.30	0.12±0.08	<0.0001
BIS	53.4±14.7	84.6±13.6	<0.0001
SEF_{95} (Hz)	17.1±4.3	22.0±4.3	<0.0001
RelativeBetaRatio	−1.54±0.24	−1.34±0.24	<0.0001
EMGlow (dB)	29.44±4.7	50.64±10.82	<0.0001
Total power (μV)			
f0: 0.5–47Hz	74.7±5.0	72.9±6.1	0.0308
f1: 0.5–8Hz	70.9±5.0	69.6±6.6	0.1587
f2: 8–13Hz	64.0±5.5	59.7±5.7	<0.0001
f3: 13–20Hz	61.0±5.8	56.9±5.6	<0.0001
f4: 20–30Hz	55.1±6.7	55.8±6.4	0.3492
f5: 30–47Hz	48.6±6.5	55.4±7.6	<0.0001
SD1/SD2			
f0: 0.5–47Hz	0.24±0.08	0.33±0.18	0.0007
f1: 0.5–8Hz	0.12±0.02	0.08±0.02	<0.0001
f2: 8–13Hz	0.24±0.04	0.20±0.05	<0.0001
f3: 13–20Hz	0.35±0.04	0.40±0.05	<0.0001
f4: 20–30Hz	0.59±0.03	0.69±0.05	<0.0001
f5: 30–47Hz	1.13±0.06	1.29±0.10	<0.0001
PP_A (μV^2)			
f0: 0.5–47Hz	491.8±644.5	462.7±685.0	0.7774
f1: 0.5–8Hz	90.2±142.4	68.0±134.2	0.3793
f2: 8–13Hz	56.2±83.3	20.2±41.9	0.0018
f3: 13–20Hz	44.3±63.4	20.0±39.4	0.0037
f4: 20–30 Hz	21.5±31.7	28.9±58.1	0.3148
f5: 30–47 Hz	5.7±8.1	65.9±167.3	0.0065
PP_{AR}			
f1: 0.5–8Hz	0.212±0.096	0.164±0.098	0.0026
f2: 8–13Hz	0.106±0.028	0.054±0.033	<0.0001
f3: 13–20Hz	0.082±0.037	0.049±0.0295	<0.0001
f4: 20–30Hz	0.039±0.036	0.057±0.036	0.0052
f5: 30–47Hz	0.012±0.020	0.100±0.096	<0.0001
PIS	55.8±5.3	79.5±13.3	<0.0001

Minimum alveolar concentration 50% (MAC) of sevoflurane and desflurane was calculated by using 1.71 and 7.25, respectively. Data of EM_{-10} and EM_0 are shown as mean±SD. Student's paired *t*-test was used for statistical comparisons between the data from two time points, EM_{-10} and EM_0. all$_{_combined}$, a total of 60 patients from three groups of patients; BIS, Bispectral index; EEG, electroencephalography; EM_{-10}, 10 min before emergence; EM_0, at the time of emergence; et_{AG} ,end-tidal anesthetic gas concentration; PIS, EMGlow, a band power for the range 70-110 Hz, value in decibel (dB) with respect to 0.0001 μV^2; Poincaré plot-area integrated score; PP_A, Poincaré plot area; PP_{AR}, Poincaré plot-area ratio; SEF_{95}, spectral edge frequency below which 95% of the power of a given signal is located.
