# Supplementary material for: Poincaré Plot Area of Gamma-Band EEG as a Measure of Emergence From Inhalational General Anesthesia
Source: Front Physiol. 2021 Feb 9;12:627088. doi: 10.3389/fphys.2021.627088 (PMC7900422; doi:10.3389/fphys.2021.627088)
Supplement: Supplementary Table 3 — Evaluation of linear regression of the three groups. [file Table_3.PDF]

SUPPLEMENTARY TABLE S3. Evaluation of linear regression of three groups.

Tab	Adult$_{SEV}$				adult$_{DES}$				ped$_{SEV}$			
x-parameter	PIS				PIS				PIS			
y-parameter	BIS				BIS				BIS			
	slope	y-intercept	R^2	RMSE	slope	y-intercept	R^2	RMSE	slope	y-intercept	R^2	RMSE
total*	0.83	12.91	0.98	2.63	0.73	21.76	0.98	8.21	0.91	8.54	0.98	2.37
1	0.94	7.15	0.89	4.57	0.55	31.43	0.87	12.38	0.55	29.14	0.82	10.03
2	0.94	5.98	0.72	4.44	0.81	17.75	0.90	9.77	0.23	67.74	0.19	10.79
3	0.33	43.59	0.30	4.87	0.63	23.78	0.63	6.18	0.38	55.23	0.30	6.17
4	0.77	13.69	0.92	4.23	0.84	12.06	0.91	5.73	0.72	23.13	0.54	4.65
5	1.47	-40.96	0.81	13.95	0.69	10.45	0.76	6.90	0.73	19.98	0.85	5.08
6	0.47	31.45	0.09	7.31	0.80	19.58	0.91	10.97	0.11	67.63	0.007	5.82
7	1.27	-23.04	0.86	9.78	0.66	-20.90	0.65	14.28	0.91	6.67	0.71	5.70
8	0.43	28.59	0.17	7.91	0.83	-13.96	0.92	6.57	1.10	-8.85	0.80	4.00
9	0.74	14.24	0.90	5.84	0.65	26.28	0.80	8.27	0.72	28.60	0.42	10.59
10	1.14	-8.52	0.79	5.26	0.62	15.94	0.40	10.92	0.22	61.47	0.09	4.66
11	0.82	10.97	0.68	3.46	0.99	8.67	0.78	10.63	0.79	16.58	0.36	5,01
12	1.13	-7,71	0.51	5.88	0.49	33.90	0.83	7.30	1.04	2.72	0.90	7.71
13	1.24	-21.34	0.86	7.19	0.39	36.13	0.39	10.95	0.89	11.52	0.88	6,10
14	0.59	26.46	0.28	5.22	0.55	30.28	0.81	10.48	1.26	-16.90	0.76	3.86
15	0.66	19.43	0.94	6.80	0.31	45.23	0.18	7.52	1.08	-7.96	0.33	9.83
16	0.79	13.59	0.55	2.98	0.61	30.65	0.86	14.39	0.48	41.93	0.83	17.84
17	1.91	-56.00	0.70	8.46	0.47	35.52	0.34	13.33	0.93	6.61	0.66	3.47
18	0.86	9.35	0.70	3.35	0.64	30.30	0.82	19.25	1.30	-21.71	0.83	4.61
19	1.11	-12.53	0.63	11.34	0.56	27.98	0.54	11.71	0.82	14.26	0.26	3.98
20	0.55	26.55	0.77	3.24	0.66	25.51	0.71	13.26	1.04	-2.66	0.80	3.59
Mean	0.91	4.67	0.65	6.30	0.64	21.33	0.70	10.54	0.77	19.76	0.57	6.80
SD	0.39	25.79	0.26	2.88	0.16	16.27	0.22	3.42	0.34	27.30	0.29	3.74

Linear correlations were calculated between an x-parameter (PIS) and a y-parameter (BIS), and the values of slope, y-intercept, and R^2 of the linear regressions and RMSE were calculated in each case. total* = time-course mean values of PIS and BIS were applied to the linear regression. adult$_{SEV}$, adult patients anesthetized with sevoflurane; adult$_{DES}$, adult patients anesthetized with desflurane; ped$_{SEV}$, pediatric patients anesthetized with sevoflurane. BIS, bispectral index; PIS, Poincaré plot-area integrated score; R^2, coefficient of determination; RMSE, root mean squared error.
