# Supplementary material for: Poincaré Plot Area of Gamma-Band EEG as a Measure of Emergence From Inhalational General Anesthesia
Source: Front Physiol. 2021 Feb 9;12:627088. doi: 10.3389/fphys.2021.627088 (PMC7900422; doi:10.3389/fphys.2021.627088)
Supplement: Supplementary Data Sheet 1 — The example codes of Poincaré plot parameters – calculations with sample data using Python in Jupyter Notebook, and Processing. [file Data_Sheet_1.PDF]

Supplementary_Method

- Calculations for Poincare Plot Indices

Python Calculations for Poincare Plots Parameters

・Import libraries (numpy, matplotlib, pyhrv) and setup svg format.

In [1]:
```
import numpy as np
import matplotlib.pyplot as plt
import pyhrv.nonlinear as nl

from IPython.display import set_matplotlib_formats
set_matplotlib_formats('svg')
```

・Create EEG data (1second μV data, 128Hz, 128 datapoints). Put the data (128 data points) to numpy array named "eeg_128".

In [2]:
```
eeg_128 = np.array([  3.65,  14.05,  23.8 ,  28.95,  26.5 ,  20.25,  17.5 ,  13.05,
          1.6 ,  -9.45, -11.2 ,  -5.15,  10.  ,  32.75,  42.6 ,  31.5 ,
         17.85,   7.4 ,  -2.5 ,  -9.  , -12.6 ,  -9.3 ,   3.45,  17.05,
         26.75,  30.45,  25.85,  24.1 ,  23.15,  15.35,   5.8 ,  -2.85,
         -7.9 ,  -4.4 ,  -1.25,   3.4 ,  19.5 ,  37.3 ,  42.5 ,  34.2 ,
         20.65,  12.1 ,   8.9 ,   7.8 ,   6.5 ,  -1.8 , -12.3 , -12.25,
         -5.25,  -1.15,  -1.35,  -1.4 ,   3.9 ,  13.9 ,  18.4 ,  12.35,
          1.85,  -5.9 ,  -8.9 ,  -7.15,   2.  ,  19.75,  33.95,  34.05,
         23.05,   8.1 ,  -4.95,  -9.  ,  -3.45,   4.65,   5.5 ,  -3.9 ,
        -18.25, -21.45,  -2.55,  29.35,  46.55,  35.7 ,   8.35, -12.7 ,
        -19.65, -16.45,  -5.  ,  13.4 ,  24.35,  20.9 ,  13.7 ,  13.85,
         15.3 ,   8.05,  -5.25, -13.4 , -13.4 ,  -6.75,   5.95,  19.1 ,
         22.5 ,  17.75,  14.9 ,  14.35,   4.3 , -13.75, -17.9 ,   0.35,
         20.95,  29.9 ,  32.  ,  28.35,  17.  ,   6.1 ,   0.45,  -4.1 ,
         -6.9 ,  -3.1 ,   5.75,  11.7 ,  13.3 ,  11.3 ,   3.7 ,  -4.9 ,
         -3.45,   9.  ,  19.7 ,  17.7 ,   3.2 , -14.35, -20.6 ,  -9.3 ])
```

・display eeg_128 data.

In [5]:
```
eeg_128
```

Out[5]:
```
array([  3.65,  14.05,  23.8 ,  28.95,  26.5 ,  20.25,  17.5 ,  13.05,
          1.6 ,  -9.45, -11.2 ,  -5.15,  10.  ,  32.75,  42.6 ,  31.5 ,
         17.85,   7.4 ,  -2.5 ,  -9.  , -12.6 ,  -9.3 ,   3.45,  17.05,
         26.75,  30.45,  25.85,  24.1 ,  23.15,  15.35,   5.8 ,  -2.85,
         -7.9 ,  -4.4 ,  -1.25,   3.4 ,  19.5 ,  37.3 ,  42.5 ,  34.2 ,
         20.65,  12.1 ,   8.9 ,   7.8 ,   6.5 ,  -1.8 , -12.3 , -12.25,
         -5.25,  -1.15,  -1.35,  -1.4 ,   3.9 ,  13.9 ,  18.4 ,  12.35,
          1.85,  -5.9 ,  -8.9 ,  -7.15,   2.  ,  19.75,  33.95,  34.05,
         23.05,   8.1 ,  -4.95,  -9.  ,  -3.45,   4.65,   5.5 ,  -3.9 ,
        -18.25, -21.45,  -2.55,  29.35,  46.55,  35.7 ,   8.35, -12.7 ,
        -19.65, -16.45,  -5.  ,  13.4 ,  24.35,  20.9 ,  13.7 ,  13.85,
         15.3 ,   8.05,  -5.25, -13.4 , -13.4 ,  -6.75,   5.95,  19.1 ,
         22.5 ,  17.75,  14.9 ,  14.35,   4.3 , -13.75, -17.9 ,   0.35,
         20.95,  29.9 ,  32.  ,  28.35,  17.  ,   6.1 ,   0.45,  -4.1 ,
         -6.9 ,  -3.1 ,   5.75,  11.7 ,  13.3 ,  11.3 ,   3.7 ,  -4.9 ,
         -3.45,   9.  ,  19.7 ,  17.7 ,   3.2 , -14.35, -20.6 ,  -9.3 ])
```

In [4]:
```
len(eeg_128)
```

Out[4]:
```
128
```

・Display eeg_128 as EEG wave.

In [7]:
```
#欠損値を補完補正した脳波データを時系列グラフ表示
fig, ax = plt.subplots(figsize=(12,4))
plt.plot(eeg_128)
```

Out[7]:
```
[<matplotlib.lines.Line2D at 0x1208ff588>]
```

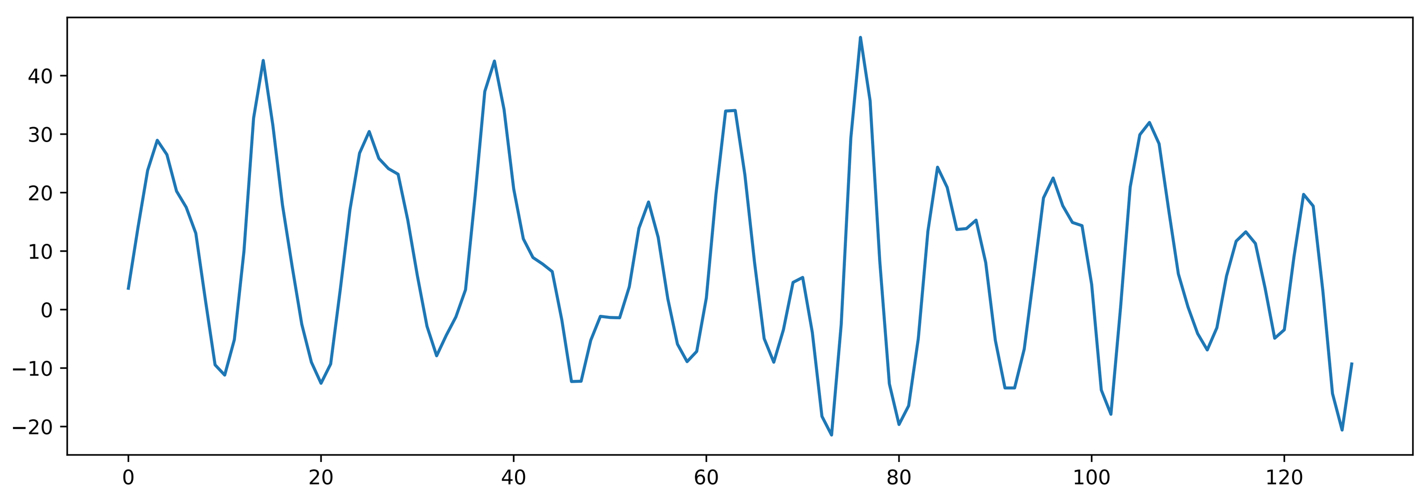

For Ponicaré Plots (xy-scatter graph), set x and y data. x data = data from #1-#127. y data = data from #2-#128.

In [8]:
```
x=eeg_128[0:127]
y=eeg_128[1:128]
```

・Display Pioncaré Plots with the linear regression line.

In [15]:
```
a, b = np.polyfit(x, y, 1)
y2 = a * x + b

fig = plt.figure(dpi=300, figsize=(5,5))

plt.scatter(x, y, alpha=0.5, color="Red", s=10, linewidth=.1)
plt.xlim(-60, 85)
plt.ylim(-60, 85)
plt.grid(True)
plt.plot(x, y2, color='blue',  linewidth=.8, linestyle="dashed")
plt.text(0.1, a*0.1+b, 'y='+str(round(a,4)) + 'x+'+str(round(b, 4)))
plt.savefig('xy_1_poincare_128.svg')
plt.show()
```

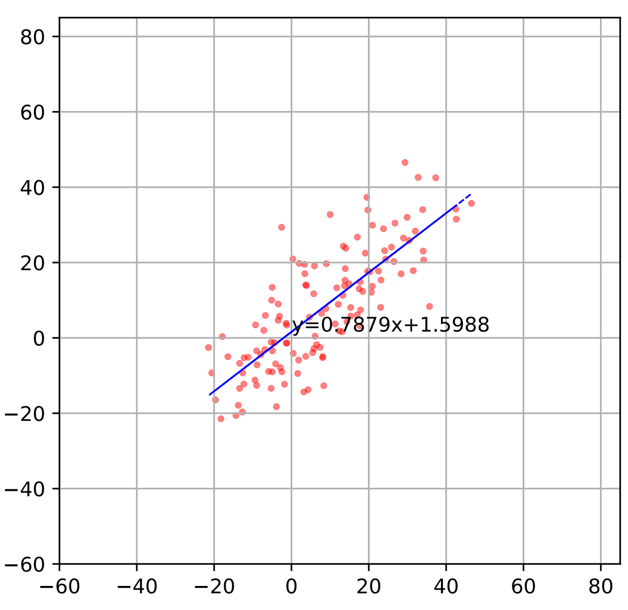

・Calculate the Pioncaré Plot Indices, and display the Pioncaré Plots using poincare() of the library pyhrv.

In [11]:
```
results = nl.poincare(eeg_128)
```

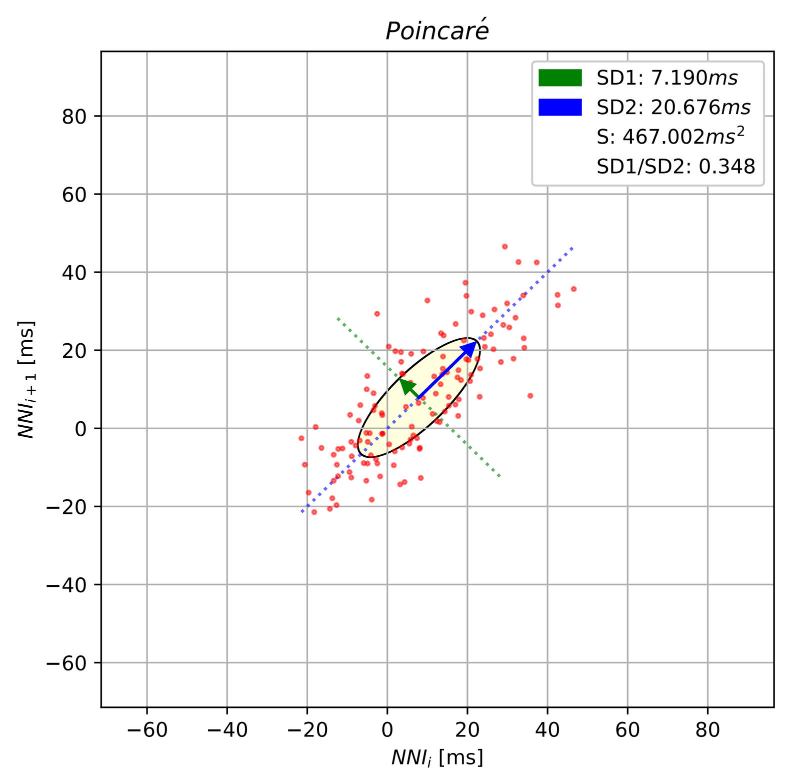

In [12]:
```
print("SD1=:", results['sd1'])
print("SD1=:",results['sd2'])
print("SD1/SD2=:",results['sd_ratio'])
print("PP_area=:",results['ellipse_area'])
```

```
SD1=: 7.189633834672621
SD1=: 20.675801813050953
SD1/SD2=: 2.875779530431736
PP_area=: 467.00228527701455
```

・Then, Pioncaré Plot area ratio is calculated by the following formula.

1) Pioncaré Plot area ratio_f1 (PP_AR_f1) = [Pioncaré Plot area at f1 (PP_A_f1)] / [Pioncaré Plot area at f0 (PP_A_f0)]

2) Pioncaré Plot area ratio_f2 (PP_AR_f2) = [Pioncaré Plot area at f2 (PP_A_f2)] / [Pioncaré Plot area at f0]

3) Pioncaré Plot area ratio_f3 (PP_AR_f3) = [Pioncaré Plot area at f3 (PP_A_f3)] / [Pioncaré Plot area at f0]

4) Pioncaré Plot area ratio_f4 (PP_AR_f4) = [Pioncaré Plot area at f4 (PP_A_f4)] / [Pioncaré Plot area at f0]

5) Pioncaré Plot area ratio_f5 (PP_AR_f5) = [Pioncaré Plot area at f5 (PP_A_f5)] / [Pioncaré Plot area at f0]

Processing Poincare Plots: function poincare()

```
//define variables
double x; //EEG microvolt data
int data_n = 128; //the number of EEG microvolt data
float[] x_sd_final; //poincare plot indice

void setup(){
//mini-sample EEG data (1 sec, 128Hz, 128 data points)
double[] x = {3.65, 14.05, 23.8, 28.95, 26.5, 20.25, 17.5 , 13.05, 1.6, -9.45, -11.2, -5.15, 10.0, 32.75, 42.6, 31.5, 17.85, 7.4, -2.5, -9.0, -12.6, -9.3, 3.45, 17.05, 26.75, 30.45, 25.85, 24.1, 23.15, 15.35, 5.8, -2.85, -7.9, -4.4, -1.25, 3.4, 19.5, 37.3, 42.5, 34.2, 20.65, 12.1, 8.9, 7.8, 6.5, -1.8, -12.3, -12.25, -5.25, -1.15, -1.35, -1.4, 3.9, 13.9, 18.4, 12.35, 1.85, -5.9, -8.9, -7.15, 2.0, 19.75, 33.95, 34.05, 23.05, 8.1, -4.95, -9.0, -3.45, 4.65, 5.5, -3.9, -18.25, -21.45, -2.55, 29.35, 46.55, 35.7, 8.35, -12.7, -19.65, -16.45, -5.0, 13.4, 24.35, 20.9, 13.7, 13.85, 15.3, 8.05, -5.25, -13.4, -13.4, -6.75, 5.95, 19.1 , 22.5 , 17.75, 14.9, 14.35, 4.3, -13.75, -17.9, 0.35, 20.95, 29.9, 32.0, 28.35, 17.0, 6.1, 0.45, -4.1, -6.9, -3.1, 5.75, 11.7, 13.3, 11.3, 3.7, -4.9, -3.45, 9.0, 19.7, 17.7, 3.2, -14.35, -20.6, -9.3};
//call function poincare()
x_sd_final = poincare(x);

//print the results: SD1, SD2, SD1/SD2, and the Poincare Plot area.
println("SD1=;", x_sd_final[0]);
println("SD2=;", x_sd_final[1]);
println("SD1/SD2=;", x_sd_final[2]);
println("area=;", x_sd_final[3]);
}

//function poincare()
float[] poincare(double[] _x) {
//define the internal variables
float x_avg; //average of EEG microvolts data.
float[] d0_x; //array for EEG microvolts data adjusted mean.
float d0_x_avg; //mean of array for EEG microvolts data adjusted mean.
float[] x_sd1; //array for sd1 calculation.
float[] x_sd2; //array for sd2 calculation.
float x_sd1_final; //SD1
float x_sd2_final; //SD2
float x_sd2_div_sd1; //SD2/SD1
float x_sd1_div_sd2; //SD1/SD2
float x_area; //the Poincare Plot area


x_avg = 0; //initialization of x_avg

d0_x = new float[128]; //initialization of d0_x
x_sd1 = new float[128]; //initialization of x_sd1
x_sd2 = new float[128]; //initialization of x_sd2


for (int i=0; i<data_n; i++) { //calculate the x_avg
x_avg += (float) _x[i];
}
x_avg = x_avg/data_n;


for (int i=0; i<data_n; i++) { //calculate the d0_x
d0_x[i] = (float) _x[i] - x_avg;
}

d0_x_avg = array_avg_float(d0_x); //calculate the average of d0_x as d0_x_avg

for (int i=0; i<data_n-1; i++) { //calculate SD1 and SD2 arrays
x_sd1[i]= sqrt((d0_x[i]-d0_x[i+1])(d0_x[i]-d0_x[i+1]))/sqrt(2);
x_sd2[i]= sqrt(((d0_x[i]+d0_x[i+1])/2 - d0_x_avg)((d0_x[i]+d0_x[i+1])/2 - d0_x_avg))*sqrt(2);
}

x_sd1_final = array_rms_float(x_sd1); // calculate SD1 from RMS of x_sd1
x_sd2_final = array_rms_float(x_sd2); // calculate SD2 from RMS of x_sd2

x_sd2_div_sd1 = x_sd2_final/x_sd1_final; //calculate SD2/SD1
x_sd1_div_sd2 = 1/ x_sd2_div_sd1; //calculate SD1/SD2
x_area = x_sd1_final x_sd2_final PI; //calculate the Poincare Plot area (PP_A)

float[] poincare = {x_sd1_final, x_sd2_final, x_sd1_div_sd2, x_area}; //set the results to the array poincare

return poincare; // retrun the results
}

float array_avg_float(float[] _array) { //function to calculate the average of array items
float _array_avg = 0;

for (int i=0; i<_array.length; i++) {
_array_avg += _array[i];
}
_array_avg = _array_avg / (float)_array.length;

return _array_avg;
}

float array_rms_float(float[] _array) { //function to calculate the RMS of array items
float _array_rms = 0;

for (int i=0; i<_array.length; i++) {
_array_rms += _array[i] * _array[i];
}
_array_rms = sqrt(_array_rms / (float) _array.length);

return _array_rms;
}
```

In [ ]:
